# Human IgG1 antibodies suppress angiogenesis in a target-independent manner

**DOI:** 10.1038/sigtrans.2015.1

**Published:** 2016-01-28

**Authors:** Sasha Bogdanovich, Younghee Kim, Takeshi Mizutani, Reo Yasuma, Laura Tudisco, Valeria Cicatiello, Ana Bastos-Carvalho, Nagaraj Kerur, Yoshio Hirano, Judit Z Baffi, Valeria Tarallo, Shengjian Li, Tetsuhiro Yasuma, Parthasarathy Arpitha, Benjamin J Fowler, Charles B Wright, Ivana Apicella, Adelaide Greco, Arturo Brunetti, Menotti Ruvo, Annamaria Sandomenico, Miho Nozaki, Ryo Ijima, Hiroki Kaneko, Yuichiro Ogura, Hiroko Terasaki, Balamurali K Ambati, Jeanette HW Leusen, Wallace Y Langdon, Michael R Clark, Kathryn L Armour, Pierre Bruhns, J Sjef Verbeek, Bradley D Gelfand, Sandro De Falco, Jayakrishna Ambati

**Affiliations:** 1 Department of Ophthalmology and Visual Sciences, University of Kentucky, Lexington, KY, USA; 2 Department of Ophthalmology and Visual Science, Nagoya City University Graduate School of Medical Sciences, Nagoya, Japan; 3 Department of Ophthalmology, Nagoya University Graduate School of Medicine, Nagoya, Japan; 4 Angiogenesis Lab, Institute of Genetics and Biophysics—CNR, Naples, Italy; 5 Bio-Ker, MultiMedica Group, Naples, Italy; 6 Department of Advanced Biomedical Sciences, University of Naples ‘Federico II’, Naples, Italy; 7 CEINGE—Biotecnologie Avanzate, s.c.a.r.l., Naples, Italy; 8 Istituto di Biostrutture e Bioimmagini, CNR, Naples, Italy; 9 Department of Ophthalmology and Visual Sciences, Moran Eye Center, University of Utah School of Medicine, Salt Lake City, UT, USA; 10 Department of Ophthalmology, Veterans Affairs Salt Lake City Healthcare System, Salt Lake City, UT, USA; 11 Immunotherapy Laboratory, Laboratory for Translational Immunology, University Medical Center Utrecht, Utrecht, The Netherlands; 12 School of Pathology and Laboratory Medicine, University of Western Australia, Crawley, WA, Australia; 13 Division of Immunology, Department of Pathology, University of Cambridge, Cambridge, UK; 14 Department of Immunology, Unit of Antibodies in Therapy and Pathology, Institut Pasteur, Paris, France; 15 Institut National de la Santé et de la Recherche Médicale (INSERM) U1222, Paris, France; 16 Department of Human Genetics, Leiden University Medical Center, Leiden, The Netherlands; 17 Department of Biomedical Engineering, University of Kentucky, Lexington, KY, USA; 18 Department of Microbiology, Immunology, and Molecular Genetics, University of Kentucky, Lexington, KY, USA; 19 IRCCS MultiMedica, Milano, Italy; 20 Department of Physiology, University of Kentucky, Lexington, KY, USA

## Abstract

Aberrant angiogenesis is implicated in diseases affecting nearly 10% of the world’s population. The most widely used anti-angiogenic drug is bevacizumab, a humanized IgG1 monoclonal antibody that targets human VEGFA. Although bevacizumab does not recognize mouse Vegfa, it inhibits angiogenesis in mice. Here we show bevacizumab suppressed angiogenesis in three mouse models not via Vegfa blockade but rather Fc-mediated signaling through FcγRI (CD64) and c-Cbl, impairing macrophage migration. Other approved humanized or human IgG1 antibodies without mouse targets (adalimumab, alemtuzumab, ofatumumab, omalizumab, palivizumab and tocilizumab), mouse IgG2a, and overexpression of human IgG1-Fc or mouse IgG2a-Fc, also inhibited angiogenesis in wild-type and FcγR humanized mice. This anti-angiogenic effect was abolished by Fcgr1 ablation or knockdown, Fc cleavage, IgG-Fc inhibition, disruption of Fc-FcγR interaction, or elimination of FcRγ-initated signaling. Furthermore, bevacizumab’s Fc region potentiated its anti-angiogenic activity in humanized VEGFA mice. Finally, mice deficient in FcγRI exhibited increased developmental and pathological angiogenesis. These findings reveal an unexpected anti-angiogenic function for FcγRI and a potentially concerning off-target effect of hIgG1 therapies.

## Introduction

Dozens of monoclonal antibodies are approved by the United States Food and Drug Administration, European Medicines Agency, and other regulatory agencies for treating numerous diseases including age-related macular degeneration (AMD), asthma, autoimmune disorders and multiple cancers. These drugs are used in millions of people worldwide with global sales exceeding $50 billion.^1^ In addition, there are hundreds of ongoing clinical trials evaluating various other monoclonal antibodies.^1^

Bevacizumab (Avastin), a humanized monoclonal IgG1 that targets VEGFA,^2^ inhibits blood vessel growth and has been approved for treating multiple cancers,^3^ and is widely used to treat neovascular AMD.^4^ Bevacizumab is exquisitely specific for human VEGFA, having no measurable binding affinity for, or ability to functionally inhibit, murine Vegfa.^5–7^ Surprisingly, numerous reports claim an anti-angiogenic effect of bevacizumab in various murine models of neovascularization.^8–14^ Yet nearly all these reports have compared bevacizumab with saline or no treatment controls rather than to a biologically appropriate human IgG1 control. We suspected, therefore, that the angioinhibitory effect of bevacizumab in murine models was misattributed to blockade of Vegfa, and was instead due to an intrinsic property of the IgG1 molecule independent of its antigenic specificity, namely a target-independent effect.

In this study, we found that bevacizumab, and numerous other therapeutic human IgG1 antibodies, as well as mouse IgG2a, suppressed angiogenesis in mice via FcγRI, the high-affinity IgG receptor.^15–17^ These effects were observed both with local and systemic administration of these antibody preparations at doses similar to or identical to those used in humans for various diseases.

A prospective randomized clinical trial reported in patients with corneal angiogenesis that bevacizumab, a full-length antibody that neutralizes human VEGFA activity and is able to bind FcγRs, is superior to ranibizumab, a humanized IgG1 Fab fragment that blocks human VEGFA but cannot bind FcγRs, in inhibiting angiogenesis.^18^ Our findings provide a molecular basis for this clinical observation. In contrast, clinical trials in patients with choroidal angiogenesis found no significant difference in the effects of bevacizumab versus ranibizumab, each tested at a single dose, on angiogenic lesion size.^4,19^ Our findings suggest that the dose of bevacizumab required to achieve FcγRI-mediated anti-angiogenic activity is roughly eight times higher than the dose used in these trials, which is sufficient only to neutralize human VEGFA, thereby providing a molecular rationale for testing such higher doses.

Angiogenic diseases collectively affect half-a-billion people;^20^ together, our data provide evidence that human IgG1 antibodies, as a class, form an important group of angioinhibitors, potentially fill the need for developing inexpensive generic human IgG1 drugs,^21^ and raise awareness for monitoring possible unintended effects on blood vessels by these widely used therapeutics. We also found increased pathological and developmental angiogenic responses in mice lacking FcγRI, suggesting that endogenous Igs also have a role in vascular patterning.

## Materials and methods

### Animals

All animal experiments were in accordance with the guidelines of the relevant institutional authorities. Male mice, aged 4–8 weeks, were randomized 1:1 to treatment with active drug versus inactive drug or control treatments.

### Corneal angiogenesis

Nylon sutures (Mani, Utsunomiya, Japan) were placed into the corneal stroma of mice, and on day 10 after injury, we calculated the mean percentage CD31^+^Lyve1^−^ blood vessel areas for corneal flat mounts with ImageJ (US National Institutes of Health, Bethesda, MD, USA) as previously reported.^22,23^

### Choroidal angiogenesis

Laser photocoagulation (OcuLight GL, IRIDEX, Mountain View, CA, USA) was performed on both eyes of mice to induce neovascularization, and on day 7 after injury, choroidal angiogenesis volumes were measured by scanning laser confocal microscopy (TCS SP5, Leica, Wetzlar, Germany) as previously reported with 0.7% FITC-conjugated Isolectin B4 (Vector Laboratories, Burlingame, CA, USA).^24,25^ For intravitreous administration in choroidal angiogenesis experiments, drugs was administered into the vitreous humor of mice using a 33-gauge double-calibre needle (Ito Corporation, Tokyo, Japan) as previously described.^26^

### Hind limb ischemia angiogenesis

Unilateral proximal femoral artery ligation was performed as previously described,^27^ and on day 7 after surgery, both anterior and posterior muscles from ischemic and non-ischemic hind limbs were collected and processed for immunohistochemical analysis for vessel quantification. Color laser Doppler analysis was also performed using a dedicated Laser Doppler Perfusion Imaging System (LDPI, PeriScan PIM II System, Perimed AB, Järfälla, Sweden).

### Statistical analyses

Choroidal angiogenesis volumes per laser lesion were compared by hierarchical logistic regression using repeated measures analysis as previously described.^28^ For other comparisons, we used the Mann–Whitney *U*-test with Bonferroni correction for statistical comparison of multiple variables. Results are expressed as mean±s.e.m. Type-I error not exceeding 0.05 was deemed significant.

## Results

### Bevacizumab and human IgG1 antibodies inhibit angiogenesis in mice

**Figure 1:**
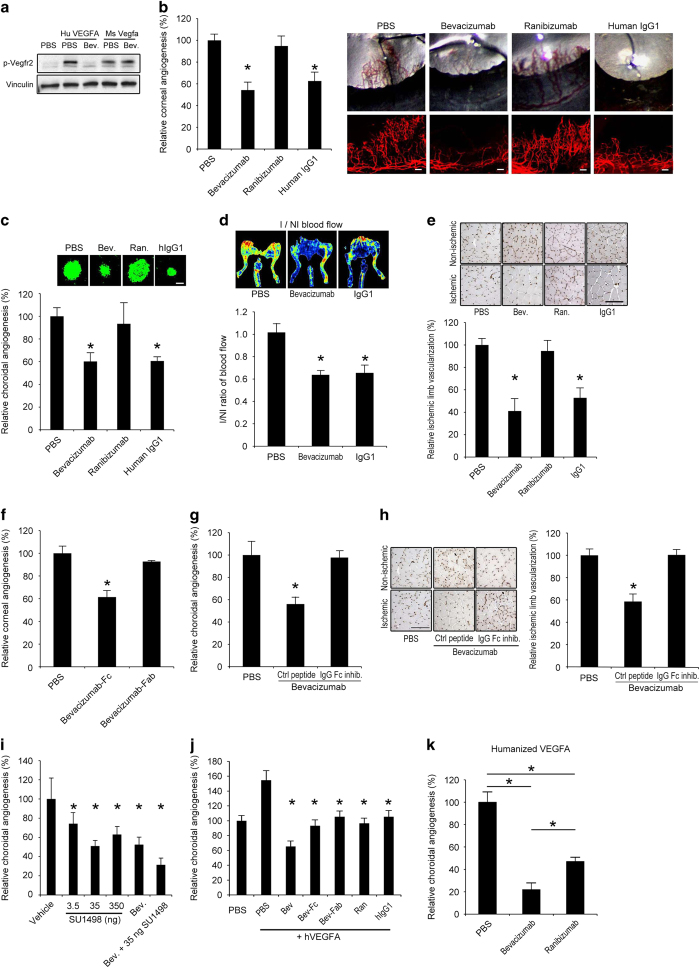
Bevacizumab inhibited mouse angiogenesis via Fc region. (**a**) Western blot shows that bevacizumab inhibited Vegfr2 phosphorylation (pVegfr2) in Py4 mouse hemangioma endothelial cells when treated with human VEGFA, but not when treated with mouse Vegfa, after 10 min. Image representative of three experiments. (**b**) Bevacizumab and human IgG1, but not ranibizumab, decreased corneal angiogenesis in wild-type mice. Area of angiogenesis was measured 10 days after suture injury and normalized to PBS group. *n*=10–38. Representative photos of wild-type mouse eyes (upper row) and corneal flat mounts (lower row) showing reduced growth of blood vessels (CD31^+^, red) in eyes treated with bevacizumab or human IgG1, but not in eyes treated with ranibizumab. Scale bars, 100 μm. (**c**) Bevacizumab and human IgG1, but not ranibizumab, suppressed choroidal angiogenesis in wild-type mice 7 days after laser injury compared with PBS (experiment performed in JA laboratory). Images depict representative choroidal angiogenesis lesions (endothelial cells stained in green) in each treatment group. *n*=12–20. (**d**, **e**) Treatment of ischemic hind limb with bevacizumab or human IgG1 in wild-type mice suppressed muscle revascularization and decreased blood vessel perfusion, as seen in representative laser Doppler perfusion images (top), and measured blood flow in the ischemic limbs (bottom), normalized to the contralateral non-ischemic limbs, 7 days after surgery. *n*=6. I/NI, ischemic/non-ischemic. Bevacizumab and human IgG1, but not ranibizumab, treatment of ischemic limbs reduced muscle angiogenesis (CD31^+^, brown) as seen in representative images of muscle CD31 immunolocalization (**e**), and quantification of muscle CD31 immunolocalization (bottom), normalized to the contralateral non-ischemic limbs. (**f**) The Fc fragments, not the Fab fragment, of bevacizumab suppressed corneal angiogenesis in wild-type mice. Area of angiogenesis was measured 10 days after suture injury and normalized to PBS group. *n*=10–38. (**g**) Co-administration of a peptide that prevents IgG-Fc binding to FcγR, but not a control peptide, blocked inhibition of choroidal angiogenesis by bevacizumab in wild-type mice. (**h**) Co-administration of an IgG-Fc inhibitory peptide, but not a control peptide, blocked inhibition of muscle angiogenesis (CD31^+^, brown) by bevacizumab, as seen in representative images of muscle CD31 immunolocalization (left), and quantification of muscle CD31 immunolocalization (right), normalized to the contralateral non-ischemic limbs. Scale bar, 100 μm. *n*=6. (**i**) Bevacizumab suppressed choroidal angiogenesis in wild-type mice to the same extent as SU1498, a small molecule tyrosine kinase inhibitor of Vegfr2. Combined administration of bevacizumab and SU1498 suppressed choroidal angiogenesis to a greater extent than either of the agents alone. *n*=6. (**j**) Choroidal angiogenesis, augmented by administration of human VEGFA, was suppressed to similar extents by ranibizumab, bevacizumab-Fab, bevacizumab-Fc and human IgG1; and, to a greater extent, by bevacizumab. *n*=6–8. (**k**) Bevacizumab suppressed choroidal angiogenesis to a greater extent than ranibizumab in the humanized VEGFA mouse, a transgenic model that expresses a VEGFA protein that can be neutralized by both bevacizumab and ranibizumab. *n*=6. Results are means±s.e.m. *P<0.05 compared with PBS (**b**–**h**, **k**) or with vehicle (**i**) or with PBS+human VEGFA (**j**).

Bevacizumab has no detectable binding to mouse Vegfa by surface plasmon resonance and does not block mouse Vegfa-induced retinal capillary endothelial cell proliferation.^5–7^ To further verify that bevacizumab does not functionally neutralize mouse Vegfa, we tested its ability to inhibit the activation of the Vegfr2 receptor tyrosine kinase in mouse Py4 hemangioma endothelial cells. As expected, bevacizumab inhibited Vegfr2 phosphorylation induced by human VEGFA but not by mouse Vegfa (Figure 1a).

We tested the effects of bevacizumab in a mouse model of suture injury-induced corneal angiogenesis, which is pathophysiologically relevant to the human condition and is driven in large part by Vegfa. We found that administration of bevacizumab into the cornea by intrastromal injection (4 μl of the commercial 25 mg/ml preparation—a dose similar to that used in humans, when corrected for relative size) inhibited corneal angiogenesis by 46% compared with phosphate-buffered saline (PBS) administration in wild-type mice (Figure 1b). This angioinhibition occurred in a dose-dependent fashion (Supplementary Figure 1). We also tested whether ranibizumab, a humanized monoclonal IgG1 Fab fragment that binds human VEGFA but not mouse Vegfa^6,29^ and is approved for the treatment of neovascular AMD, was anti-angiogenic in this model. We confirmed that, like bevacizumab, ranibizumab inhibited Vegfr2 phosphorylation induced by human VEGFA but not by mouse Vegfa (Supplementary Figure 2). However, unlike bevacizumab, ranibizumab, at equimolar amounts, did not inhibit corneal angiogenesis (Figure 1b). Interestingly, the control isotype human IgG1 for bevacizumab also reduced corneal angiogenesis in wild-type mice in a dose-dependent fashion (Figure 1b and Supplementary Figure 3).

We next tested a mouse model of laser injury-induced choroidal angiogenesis, a widely used model of neovascular AMD that is driven in large part by Vegfa and has been predictive of the success of anti-VEGFA therapies in humans. Independent testing by three different laboratories (JA, YO and HT) determined that intraocular administration of bevacizumab (1 μl of the commercial 25 mg/ml preparation—a dose approximately eight times that was used in humans, when corrected for relative size) by intravitreous injection inhibited choroidal angiogenesis by 40–45% in wild-type mice compared with PBS administration, whereas an equimolar amount of ranibizumab did not do so (Figure 1c and Supplementary Figure 4). Similar to the corneal model, the isotype human IgG1 and human IgG1-Fc also suppressed choroidal angiogenesis in wild-type mice (Figure 1c and Supplementary Figure 4).

We tested the effect of bevacizumab in a third model of angiogenesis, induced by hind limb ischemia produced by femoral artery ligation, which is a model of peripheral arterial disease. Intramuscular injection of either bevacizumab or isotype control human IgG1 suppressed limb revascularization and diminished perfusion, as monitored by color laser Doppler imaging, compared with PBS injection (Figure 1d). There was a corresponding reduction in angiogenesis by 47–59% in the bevacizumab- or human IgG1-treated limbs compared with the PBS-treated limbs, whereas ranibizumab did not suppress angiogenesis (Figure 1e). These data support the concept that human IgG1 antibodies can suppress angiogenesis in multiple tissues.

### Fc portion of human IgG1 critical for angioinhibition

Since bevacizumab and ranibizumab had nonsynonymous effects on angiogenesis in these mouse models, we suspected that the anti-angiogenic action of bevacizumab was due not to Vegfa neutralization but rather to IgG1-Fc-mediated effects. We confirmed that angioinhibition was due to the Fc, and not Fab, portion of bevacizumab by administering separately its Fab and Fc fragments as prepared from papain enzymatic digestion of the antibody (Supplementary Figure 5). Bevacizumab-Fab, but not bevacizumab-Fc, inhibited VEGFA-induced Vegfr2 phosphorylation, consistent with the location of the VEGFA-binding residues on the Fab fragment and indicating that the VEGFA neutralizing properties of bevacizumab were not affected by enzymatic digestion (Supplementary Figure 5). In contrast, bevacizumab-Fc, but not bevacizumab-Fab, reduced corneal angiogenesis in wild-type mice (Figure 1f), indicating that bevacizumab’s angioinhibitory activity in mice is due to its Fc domain and not because of binding Vegfa.

Human IgG1 and human IgG1-Fc also suppressed choroidal angiogenesis in wild-type mice (Figure 1c and Supplementary Figure 4). A peptide that prevents the binding of IgG to FcγRs by interacting with the Fc portion of IgG (IgG-Fc peptide inhibitor),^30^ but not a control peptide, eliminated the ability of bevacizumab to inhibit choroidal and hind limb angiogenesis in wild-type mice (Figure 1g and h), confirming a role for FcγR in these models.

We sought to determine whether human IgG1s would suppress angiogenesis not only when exogenously administered but also if produced endogenously. Therefore, we performed *in vivo* transfection of a plasmid encoding human IgG1-Fc coupled to an IL2-secretory sequence (phIgG1-Fc) by injecting it into the subretinal space of wild-type mice before laser injury. We found that phIgG1-Fc reduced choroidal angiogenesis in wild-type mice compared with a control null plasmid (Supplementary Figure 6). These data show that angiogenesis can be suppressed not only by exogenous administration of human IgG1 antibodies but also by endogenous overexpression of their Fc region.

### Bevacizumab and human IgG1 antibodies inhibit angiogenesis in humanized VEGF mice

Ranibizumab, which targets human VEGFA and does not have an Fc region, is effective in treating neovascular AMD in humans.^31,32^ We sought to compare the relative anti-angiogenic efficacies of *bona fide* VEGFA targeting and of FcγR-mediated signaling. First we found that the extent of suppression of choroidal angiogenesis by bevacizumab (48%) in wild-type mice was similar to that achieved by SU1498 (49%), a small molecule tyrosine kinase inhibitor of Vegfr2 (Figure 1i), and to that achieved by various inhibitors of Vegfa reported in previous studies (~40–55%).^28,33–36^ We also found that bevacizumab potentiated the angioinhibitory effects of SU1498 (Figure 1i).

Next we used a model in which laser injury-induced choroidal angiogenesis is augmented by prior intravitreous administration of human VEGFA.^37^ In this model where angiogenesis is driven both by human VEGFA and endogenous mouse pathways, we found that ranibizumab and bevacizumab-Fab, which target human VEGFA but do not contain an Fc region, suppressed angiogenesis to a similar extent as bevacizumab-Fc and human IgG1, which contain an Fc region but do not target human VEGFA (Figure 1j). In addition, full-length bevacizumab suppressed angiogenesis to a greater extent than any of the pure anti-VEGFA or Fc-containing agents alone, further indicating that modulating these two anti-angiogenic pathways simultaneously can be additive. Next, we studied ‘humanized VEGFA’ mice,^6^ wherein the mouse *Vegfa* gene was mutated such that its protein product could be neutralized by bevacizumab and ranibizumab. In this model, we found that bevacizumab suppressed choroidal angiogenesis to a significantly greater extent than ranibizumab (Figure 1k). Collectively these data demonstrate that the Fc region of bevacizumab can potentiate its anti-angiogenic effect in systems where human VEGFA is present.

### FcγRI necessary for human IgG1-induced angioinhibition

**Figure 2:**
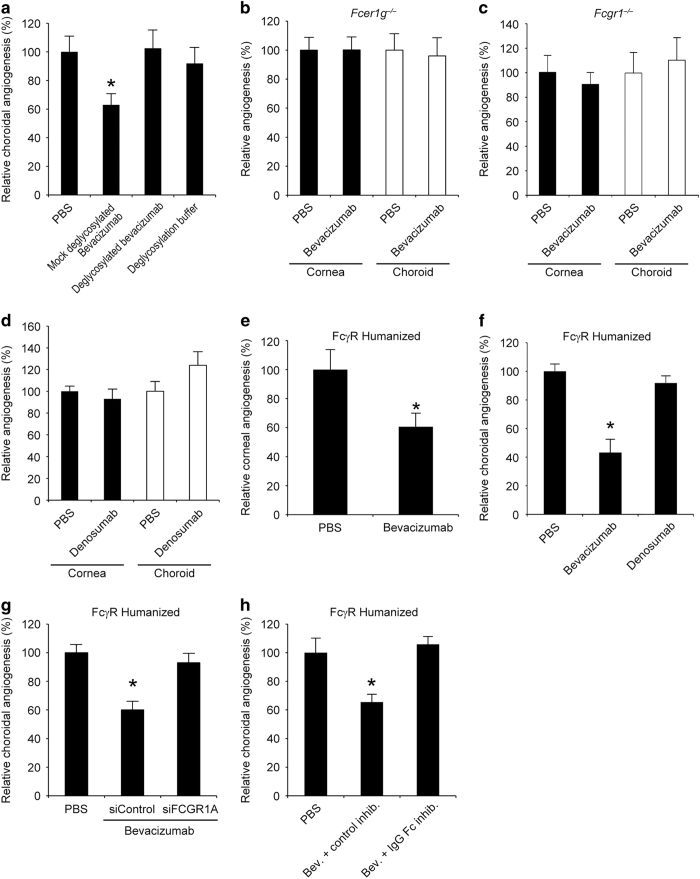
Bevacizumab inhibited mouse angiogenesis via FcγRI. (**a**) Deglycosylated bevacizumab did not suppress choroidal angiogenesis in wild-type mice; however, choroidal angiogenesis was inhibited by bevacizumab subjected to mock treatment. The deglycosylation buffer had no effect on choroidal angiogenesis. *n*=8–14. (**b**) Bevacizumab did not suppress corneal or choroidal angiogenesis in *Fcer1g*^*−/−*^ mice. *n*=8–10. (**c**) Bevacizumab did not inhibit corneal or choroidal angiogenesis in *Fcgr1*^*−/−*^ mice. No significant difference between groups. *n*=10–13. (**d**) Denosumab did not suppress corneal or choroidal angiogenesis in wild-type mice. *n*=6–8. No significant difference between groups. (**e**) Bevacizumab inhibited corneal angiogenesis in FcγR humanized mice. *n*=8. Results are means±s.e.m. *P<0.05 compared with PBS. (**f**) Bevacizumab, but not denosumab, inhibited choroidal angiogenesis in FcγR humanized mice. *n*=6–8. (**g**) Co-administration of a 17+2-nt cholesterol conjugated human *FCGR1A* siRNA, but not a 17+2-nt cholesterol-conjugated control *Luc* siRNA, blocked inhibition of choroidal angiogenesis by bevacizumab in FcγR humanized mice. *n*=8. (**h**) Co-administration of an IgG-Fc inhibitory peptide, but not a control peptide, blocked inhibition of choroidal angiogenesis by bevacizumab in FcγR humanized mice. *n*=8. Results are means±s.e.m. *P<0.05 compared with PBS (**a**, **e**–**h**).

We performed additional experiments to investigate the nature of the Fc-mediated anti-angiogenic effect of bevacizumab. It is known that deglycosylation of human IgG1 dramatically reduces its binding to both human and mouse FcγRs.^38–40^ We found that deglycosylated bevacizumab, despite retaining its ability to inhibit human VEGFA-induced Vegfr2 phosphorylation (Supplementary Figure 7), did not reduce choroidal angiogenesis in wild-type mice (Figure 2a). These data suggest that the anti-angiogenic effect of bevacizumab in mice is mediated by an endogenous FcγR that binds human IgG1.^40^

We found that bevacizumab did not suppress choroidal angiogenesis in *Fcer1g*^−/−^ (a.k.a. FcRγ^−/−^) mice (Figure 2b), which lack the common gamma chain of the activating FcγRs: FcγRI, FcγRIII and FcγRIV. To determine which activating FcγR was responsible, we tested mice lacking these receptors. First, we tested the involvement of FcγRI (encoded by *Fcgr1*), and found that bevacizumab failed to inhibit corneal or choroidal angiogenesis in *Fcgr1*^−/−^ mice (Figure 2c). In contrast, bevacizumab inhibited corneal and choroidal angiogenesis in mice lacking *Fcgr3* (Supplementary Figure 8), which encodes FcγRIII, and in mice lacking *Fcgr4* (Supplementary Figure 9), which encodes FcγRIV. Supporting the latter result, bevacizumab did not inhibit angiogenesis in *Fcgr1*^*−/−*^; *Fcgr2b*^*−/−*^; *Fcgr3*^*−/−*^; *Fcer1a*^*−/−*^ and *Fcer2a*^*−/−*^ mice, which express FcγRIV but not any of the other IgG or IgE receptors^41^ (Supplementary Figure 10). Human IgG2 binds to mouse FcγRII and FcγRIII, but not to FcγRI.^40^ The human IgG2 denosumab (Prolia: anti-RANKL) did not inhibit corneal or choroidal angiogenesis in wild-type mice (Figure 2d), suggesting that binding to FcγRI is required for IgG-induced angioinhibition.

We also found that subretinal transfection of a plasmid encoding a mutant form of human IgG1-Fc engineered with point mutations that eliminate binding to FcγRI or of a plasmid encoding human IgG2-Fc did not suppress choroidal angiogenesis in wild-type mice (Supplementary Figure 11), further supporting the concept that angioinhibition is a target-independent class effect of human or humanized IgG1 monoclonal antibodies that is mediated via FcγRI.

Administration of bevacizumab by i.v. injection every other day (to account for the 6.5-fold higher serum clearance rate in mice compared with humans,^42^ in whom it is administered weekly or every other week) also suppressed choroidal angiogenesis in wild-type mice in a dose-dependent fashion, but did not do so in *Fcgr1*^−/−^ mice (Supplementary Figure 12). Collectively, these data indicate that bevacizumab reduces mouse angiogenesis via FcγRI and not via Vegfa inhibition.

### Bevacizumab reduces angiogenesis in FcγR humanized mice

Although human IgG1 binds both mouse FcγRI and human FcγRI,^40^ the structural diversity and unique cellular expression patterns of mouse and human FcγRs are not synonymous.^43^ The generation of an FcγR humanized mouse via transgenic expression of the entire human FcγR family, under the control of their human regulatory elements, on a genetic background lacking all mouse FcγRs has enabled better prediction of the functional consequences of engaging human FcγRs by IgGs.^44^ In these FcγR humanized mice, we found that intracorneal bevacizumab reduced corneal angiogenesis just as in wild-type mice (Figure 2e). Bevacizumab also reduced choroidal angiogenesis in FcγR humanized mice whereas the human IgG2 denosumab did not (Figure 2f). As human IgG2 can bind human FcγRII and human FcγRIII but not human FcγRI,^45^ this result supports the notion that interaction with human FcγRI is mandatory for the anti-angiogenic effect of IgGs in our models. The angioinhibitory effect of bevacizumab was blocked by both the IgG-Fc peptide inhibitor and a cholesterol-conjugated^28^ *FCGR1A* siRNA (Figure 2g and h). These data demonstrate that target-independent FcγRI-mediated angioinhibitory activity of humanized monoclonal IgG1 antibodies is operative in a FcγR humanized system.

### Bevacizumab interacts with FcγRI and initiates signaling *in vivo*

**Figure 3:**
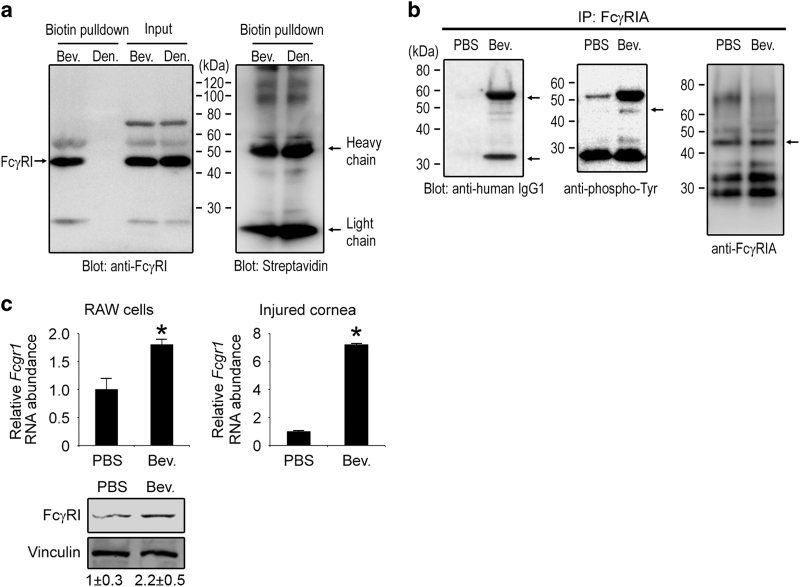
Bevacizumab interacted with, induced phosphorylation of, and upregulated abundance of FcγRI *in vivo*. (**a**) Wild-type mouse corneas that had been administered biotinylated bevacizumab or biotinylated denosumab following suture injury were subjected to streptavidin pull-down and immunoblotting for mouse FcγRI. Biotinylated bevacizumab, but not denosumab, interacted with mouse FcγRI *in vivo*. Anti-streptavidin immunoblotting confirmed efficient pull-down of both biotinylated antibodies. (**b**) FcγR humanized mouse corneas that had been administered bevacizumab or PBS following suture injury were subjected to immunoprecipitation of human FcγRI followed by immunoblotting for human IgG1 or phosphotyrosine. Bevacizumab, but not PBS, interacted with and induced phosphorylation of human FcγRI *in vivo*. Reprobing confirmed efficient immunoprecipitation of human FcγRI in both bevacizumab- and PBS-treated corneas. Each image is representative of three experiments (**a**, **b**). (**c**) Bevacizumab, but not PBS, increased *Fcgr1* mRNA abundance in RAW264.7 mouse macrophages and in wild-type mouse corneas following suture injury, as monitored by real-time reverse transcription PCR, and FcγRI protein abundance in RAW264.7 cells, as monitored by western blotting. Densitometry of FcγRI normalized to Vinculin shown. *n*=4–6. Results are means±s.e.m. *P<0.05 compared with PBS.

As we found that bevacizumab suppressed angiogenesis via FcγRI, we tested whether bevacizumab binds FcγRI *in vivo* using two complementary strategies. First, using a pull-down assay, we found that biotinylated bevacizumab, but not denosumab, that was injected into wild-type mouse corneas following suture injury co-precipitated with mouse FcγRI (Figure 3a). Next, we injected unlabeled bevacizumab into the corneas of FcγR humanized mice that were subjected to suture injury, and found that immunoprecipitation of human FcγRI pulled down human IgG1 (Figure 3b). Collectively, these data demonstrate an *in vivo* interaction between bevacizumab and both human and mouse FcγRI. In addition, bevacizumab injected into the corneas of FcγR humanized mice following suture injury-induced FcγRI phosphorylation (Figure 3b).

Crosslinking of FcγRI by human IgG1 aggregates can activate FcγRI. However, we found, using dynamic light scattering, no evidence of aggregation of bevacizumab at the administered dose (Supplementary Figure 13), as might be expected from a clinical grade preparation. This suggests that monomeric bevacizumab can induce FcγRI-mediated signaling *in vivo* in the systems we studied. Indeed, monomeric IgG engagement of other activating FcγRs has been shown to induce phosphorylation and signaling.^46,47^ Nevertheless, we cannot exclude the possibility that once the non-aggregated liquid formulation is administered into the mouse, bevacizumab might undergo *in vivo* aggregation. However, this seems unlikely given the lack of any known mouse ligand for bevacizumab. Moreover, if such *in vivo* aggregation of bevacizumab occurred in the mouse, it would also be expected to occur in human eyes because of the similar dose injected and the presence of a *bona fide* ligand—human VEGFA.

We next wondered how bevacizumab could bind FcγRI given the high serum concentration of endogenous mouse IgG that might be expected to compete for binding. Indeed, it has been shown that FcγR can still bind IgG under serum conditions^48,49^ and can execute numerous biological functions *in vivo* in response to exogenous mouse IgG2a and human IgG1 antibodies.^50–58^ This ability of FcγRI to contribute to biological signaling has been attributed to the short half-life of the interaction of FcγRI with its ligand (turnover within minutes), *de novo* synthesis of free FcγRI, receptor reorganization or conformational changes on the membrane, sampling IgG as a scavenger receptor, and ‘inside-out’ stimulation by cytokines that rapidly increases the binding of FcγRI to exogenous monomeric IgG.^48,49,51,56,59,60^ Indeed we found that bevacizumab increased FcγRI levels in mouse macrophages and in wild-type mouse corneas following suture injury (Figure 3c).

More importantly, we found that the concentrations of endogenous mouse IgG2c (an allelic variant of mouse IgG2a that is expressed in C57BL/6 mice^61,62^) in the extravascular portion of the injured tissues are minute compared with circulating levels and far lower than the extravascular tissue concentrations of exogenously administered bevacizumab (Supplementary Figure 14a and b). This paucity of extravascular mouse IgG (Supplementary Figure 14c) and the excess of bevacizumab in the injured tissues combined with increased FcγRI abundance can explain the ability of the exogenous human IgG1 to bind FcγRI *in vivo* on extravascular cells, e.g., macrophages, which express FcγRI^63^ and can modulate angiogenesis,^64^ and initiate signaling.

### Numerous therapeutic human IgG1s inhibit angiogenesis via FcγRI

**Figure 4:**
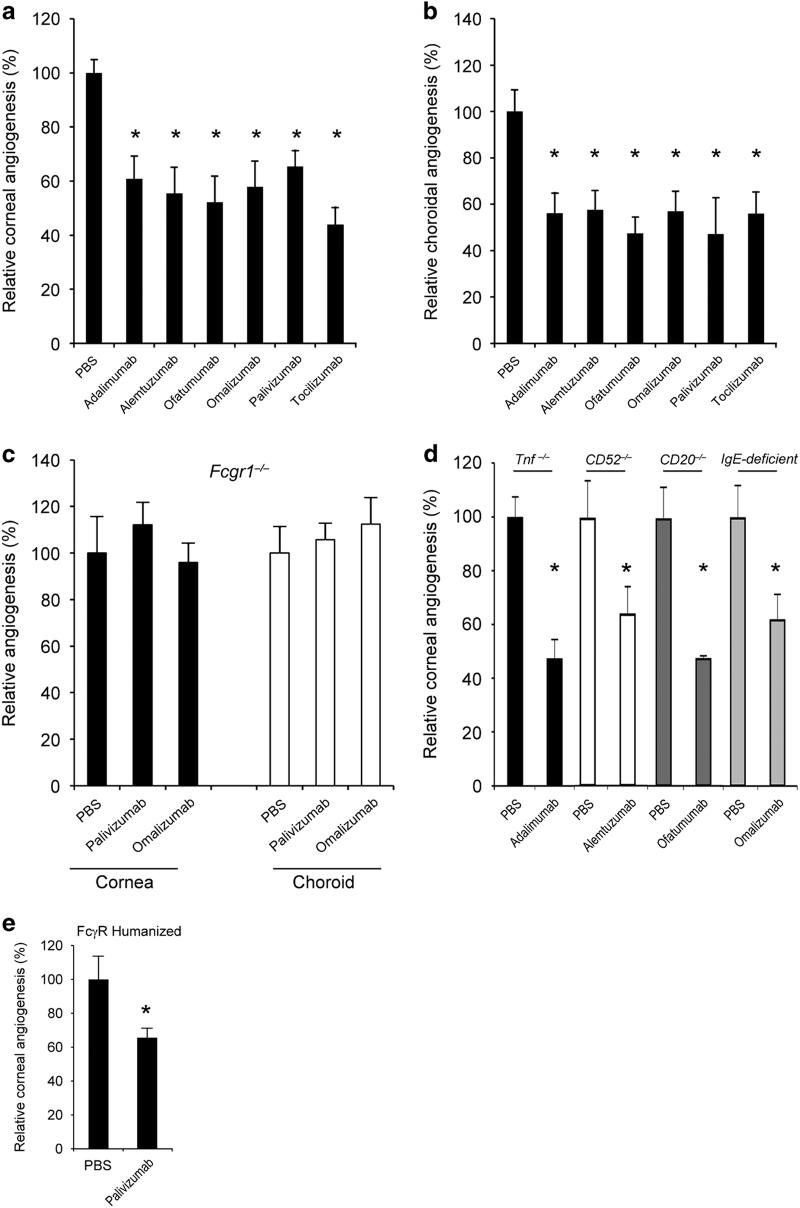
Human IgG1s inhibited mouse angiogenesis via FcγRI. Treatment with the human IgG1 antibodies adalimumab, alemtuzumab, ofatumumab, omalizumab, palivizumab or tocilizumab reduced (**a**) corneal and (**b**) choroidal angiogenesis in wild-type mice. *n*=8–19. (**c**) Palivizumab and Omalizumab did not inhibit corneal or choroidal angiogenesis in *Fcgr1*^*−/−*^ mice. *n*=6–8. No significant difference between groups. (**d**) Adalimumab, a human anti-TNFα monoclonal antibody, inhibited corneal angiogenesis in *Tnf*^*−/−*^ mice. *n*=9. Alemtuzumab, a humanized anti-CD52 monoclonal antibody, inhibited corneal angiogenesis in *CD52*^*−/−*^ mice. *n*=8. Ofatumumab, a human anti-CD20 monoclonal antibody, inhibited corneal angiogenesis in *CD20*^*−/−*^ mice. *n*=8. Omalizumab, a humanized anti-IgE monoclonal antibody, inhibited corneal angiogenesis in IgE-deficient mice. *n*=10. (**e**) Palivizumab inhibited choroidal angiogenesis in FcγR humanized mice. Results are means±s.e.m. *P<0.05 compared with PBS (**a**, **b**, **d**, **e**).

Next we assessed the anti-angiogenic effects of several human or humanized IgG1 monoclonal antibodies that are approved for treatment of various human diseases, and either do not bind the mouse homologs of their intended human protein targets or have no mammalian target: adalimumab (Humira: anti-TNFα), alemtuzumab (Campath: anti-CD52), ofatumumab (Arzerra: anti-CD20), omalizumab (Xolair: anti-IgE), palivizumab (Synagis: anti-respiratory syncytial virus protein F), and tocilizumab (Actemra: anti-IL-6R). All of these human IgG1 antibodies reduced both corneal and choroidal angiogenesis in wild-type mice (Figure 4a and b) in contrast to the human IgG2 denosumab (Figure 2e). We tested two of these antibodies—omalizumab and palivizumab—in *Fcgr1*^−/−^ mice, and found that they did not suppress corneal or choroidal angiogenesis (Figure 4c). We also found that a mutant version of alemtuzumab (G1Δab), which was engineered with point mutations in the CH2 domain of its Fc region that eliminate binding to FcγRI and reduce binding to other FcγRs,^65^ yet retains binding to human CD52 (Supplementary Figure 15a and b), did not inhibit corneal or choroidal angiogenesis in wild-type mice (Supplementary Figure 15d and e). Conversely, we found that another mutant version of alemtuzumab (D270A), which preserves binding to FcγRI but not to FcγRII and FcγRIII,^66,67^ suppressed choroidal angiogenesis in wild-type mice but not in *Fcgr1*^−/−^ mice (Supplementary Figure 15f).

We sought to exclude the possibility that the observed angioinhibition could be due to unforeseen or illegitimate interaction between these human antibodies and the mouse homologs of their human protein targets by testing them in mice deficient for the homologous genes. Such interactions were not responsible for the angiosuppression as we found that corneal angiogenesis was inhibited by adalimumab in *Tnf*^−/−^ mice, alemtuzumab in *CD52*^−/−^ mice, ofatumumab in *CD20*^−/−^ mice and omalizumab in IgE-deficient mice (Figure 4d), just as in wild-type mice. Collectively, these data indicate that multiple therapeutic human IgG1 antibodies can suppress angiogenesis via FcγRI and independent of their intended target.

Next we tested some of these antibodies in FcγR humanized mice. We found that intracorneal palivizumab reduced corneal angiogenesis (Figure 4e). In addition, alemtuzumab, but not alemtuzumab G1Δab, which does not bind FcγRI, suppressed choroidal angiogenesis in FcγR humanized mice (Supplementary Figure 16). These data demonstrate that target-independent angioinhibitory activity of humanized monoclonal IgG1 antibodies is operative in a FcγR humanized system.

### Mouse IgG2a and mouse IgG2c inhibit angiogenesis via FcγRI

To determine whether antibodies potentially produced by mice against human IgGs might have a role in the angioinhibition we observed, we tested *Rag2*^−/−^ mice, which lack B and T cells and are devoid of Igs. Bevacizumab inhibited corneal and choroidal angiogenesis in *Rag2*^−/−^ mice (Supplementary Figure 17), indicating that such an immune response potentially mounted against bevacizumab is not responsible for its angioinhibitory effect.

To exclude other potential cross-species biological effects, we tested mouse IgG2a, which like human IgG1 binds to FcγRI with high affinity.^17,41,57,68,69^ Intracorneal or subretinal transfection of a plasmid encoding mouse IgG2a-Fc coupled to an IL2-secretory sequence inhibited corneal or choroidal angiogenesis, respectively, in wild-type mice (Supplementary Figure 18). In contrast, a plasmid encoding a mutant form of mouse IgG2a-Fc engineered with point mutations that eliminate binding to FcγRI and coupled to the same IL2-secretory sequence, did not suppress angiogenesis (Supplementary Figure 18). Recombinant mouse IgG2a-Fc inhibited choroidal angiogenesis in wild-type mice in a dose-dependent fashion, whereas mouse IgG2b-Fc, which has high binding affinity for FcγRIV but not for FcγRI,^17,41,43,68,69^ did not suppress angiogenesis (Supplementary Figure 19). In addition, neither recombinant mouse IgG2a-Fc nor a plasmid encoding mouse IgG2a-Fc reduced angiogenesis in *Fcgr1*^−/−^ mice (Supplementary Figure 20). Further, mouse IgG2c also suppressed choroidal angiogenesis in wild-type mice but not in *Fcgr1*^−/−^ mice (Supplementary Figure 21). Together these data further support the concept that suppression of angiogenesis via FcγRI is not limited to human IgG1 but also is a property of mouse IgG2a and mouse IgG2c.

### Host Igs modulate angiogenesis

**Figure 5:**
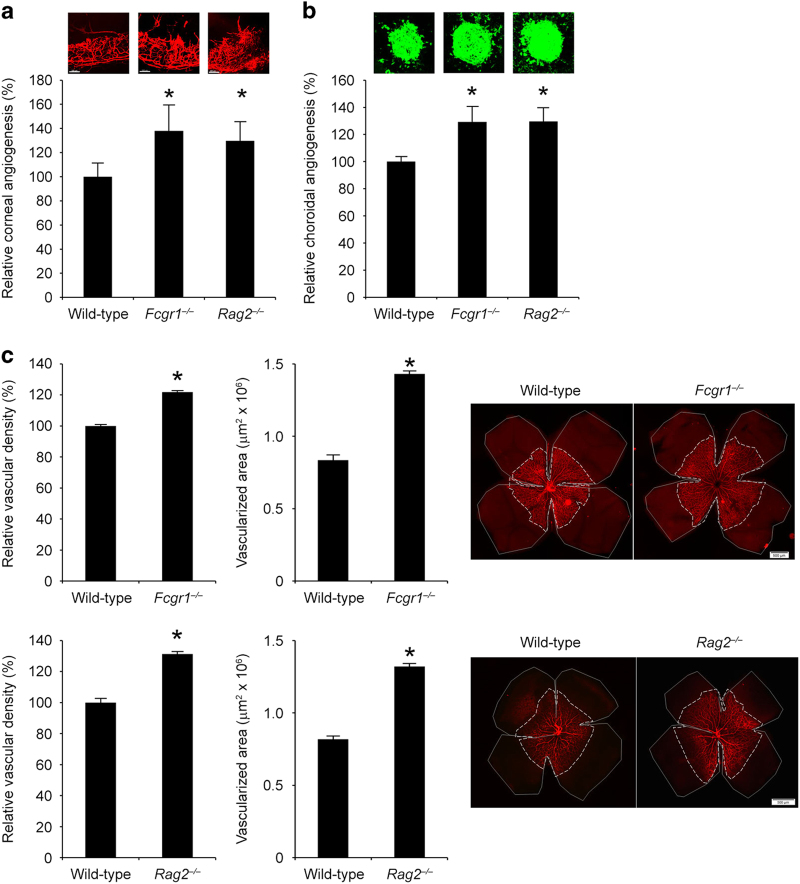
Endogenous Igs suppressed mouse angiogenesis. Corneal angiogenesis area (**a**) and choroidal angiogenesis volume (**b**) are greater in *Fcgr1*^*−/−*^ and *Rag2*^*−/−*^ mice compared with wild-type mice. *n*=8–20. (**c**) The vascular density and total area of vascularized retina at postnatal day 4 is greater in *Fcgr1*^*−/−*^ and *Rag2*^*−/−*^ mice compared with wild-type mice. *n*=8. Results are means±s.e.m. *P<0.05 compared to wild-type mice (**a**–**c**). Vascular density in the retina is normalized to wild-type mice. Representative flat mounts of corneal (**a**, red), choroidal (**b**, green) and retinal (**c**, red), vessels are shown.

As we found that recombinant and endogenously over-expressed mouse IgG2a and mouse IgG2c suppressed injury-induced angiogenesis, we explored whether native host Igs modulate vascularization. Indeed, we found that corneal and choroidal angiogenesis responses to suture or laser injury (without administration of bevacizumab), respectively, were higher in *Fcgr1*^−/−^ and *Rag2*^−/−^ mice compared with littermate wild-type controls (Figure 5a and b). Physiological vascularization of the retina during development proceeds from the central optic nerve to the periphery. This process is not complete in mice until several days after birth. We found that at postnatal day 4, both the area of vascularized retina and density of retinal vessels were greater in *Fcgr1*^−/−^ and *Rag2*^−/−^ mice compared with littermate wild-type controls (Figure 5c). Taken together, these data suggest an anti-angiogenic role for endogenous Igs in vascular patterning both during development and response to injury that is mediated via FcγRI.

### Human IgG1 reduces angiogenesis via bone marrow-derived cells expressing FcγRI

**Figure 6:**
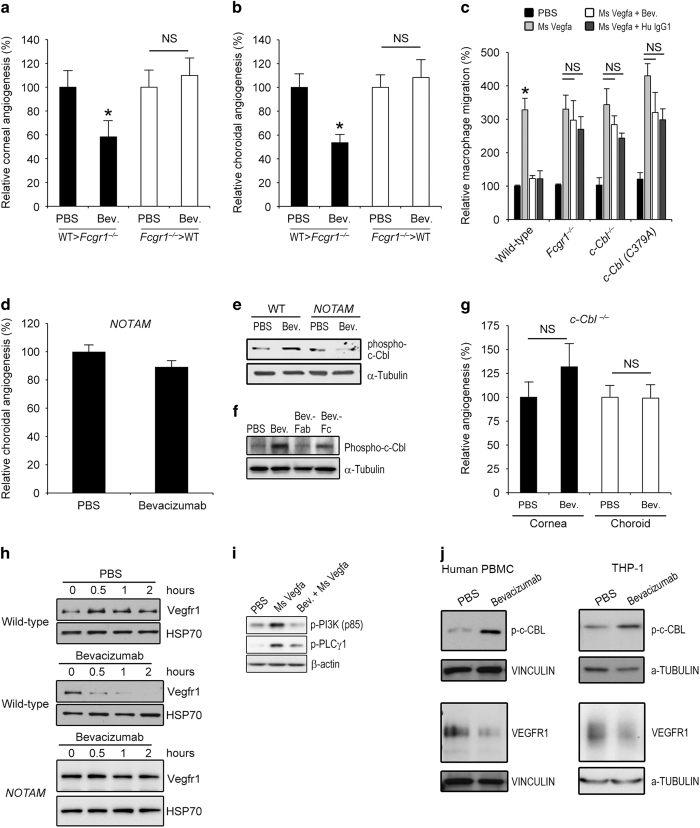
Bevacizumab inhibited angiogenesis via macrophage FcγRI and c-Cbl. Bevacizumab suppressed corneal (**a**) and choroidal (**b**) angiogenesis in *Fcgr1*^*−/−*^ mice transplanted with wild-type mouse bone marrow, but not in wild-type mice receiving *Fcgr1*^*−/−*^ bone marrow. *n*=11–16. (**c**) Bevacizumab and human IgG1 inhibited mouse Vegfa-induced migration, over 12 h, of bone marrow-derived macrophages isolated from wild-type mice but not from *Fcgr1*^*−/−*^, *c-Cbl*^*−/−*^ or c-Cbl (C379A) mice, which lack E3 ubiquitin ligase activity. *n*=3. Results are means±s.e.m. *P<0.05 compared with PBS (**a**–**c**). (**d**) Bevacizumab did not inhibit choroidal angiogenesis in NOTAM mice. *n*=10. (**e**) Western blot shows induction of c-Cbl phosphorylation in wild-type mouse BMDMs treated with bevacizumab for 15 min. Protein loading was assessed by α-Tubulin abundance. (**f**) Western blot shows *in vivo* induction of c-Cbl phosphorylation in wild-type mouse corneas following suture injury that were treated with bevacizumab or its Fc fragment, but not by its Fab fragment. (**g**) Bevacizumab did not inhibit corneal or choroidal angiogenesis in *c-Cbl*^*−/−*^ mice. *n*=11–30. NS, no significant difference between groups. (**h**) Western blots show time-dependent Vegfr1 degradation in wild-type but not NOTAM mouse BMDMs treated with bevacizumab. Protein loading was assessed by HSP70 abundance. (**i**) Western blots show that RAW264.7 mouse macrophages pre-treated with bevacizumab, but not PBS, 2 h before stimulation with mouse Vegfa, exhibited reduced phosphorylation of PI3K and PLCγ1 at 10 min after Vegfa exposure. Protein loading was assessed by β-actin abundance. (**j**) Western blots show induction of c-Cbl phosphorylation in human peripheral blood mononuclear cells (PBMC) or in THP-1 human monocytic cells, treated with bevacizumab, but not PBS, for 15 min. Treatment with bevacizumab, but not PBS, reduced VEGFR1 abundance in human PBMCs and THP-1 cells. Protein loading was assessed by Vinculin or α-Tubulin abundance. Images representative of three experiments (**e**, **f**, **h**–**j**).

To determine whether bone marrow-derived or resident cell expression of FcγRI was the critical effector in IgG1 mAb-mediated angioinhibition, we created bone marrow chimeric mice. Bevacizumab suppressed corneal and choroidal angiogenesis in *Fcgr1*^−/−^ mice receiving wild-type bone marrow but did not do so in wild-type mice receiving *Fcgr1*^−/−^ bone marrow (Figure 6a and b). These results suggest that FcγRI in bone marrow-derived cells is critical for bevacizumab-induced angioinhibition.

Among the various types of bone marrow-derived cells, macrophages are best known to have a critical role in angiogenesis.^70^ Both bevacizumab and human IgG1 inhibited mouse Vegfa-induced migration of wild-type mouse bone marrow-derived macrophages (BMDMs) but not of *Fcgr1*^−/−^ BMDMs (Figure 6c). Corroborating these data, we found that bevacizumab reduced the infiltration of F4/80+ macrophages into the sutured cornea, laser-injured choroid, and ischemic hind limb of wild-type mice (Supplementary Figure 22). These findings are in concert with the abundant expression of FcγRI by macrophages.^63,71^

We next assessed whether bevacizumab induces intracellular FcγR-mediated signaling events. First we tested the FcRγ-chain signaling-deficient NOTAM mice, which exhibit normal cell surface Fc receptor expression and normal IgG binding, but have non-signaling Fc receptors because their associated γ-chains have been mutated in their immunoreceptor tyrosine-based activation motif, which is responsible for signal transduction.^72^ We found that bevacizumab did not suppress choroidal angiogenesis in FcR NOTAM mice (Figure 6d), suggesting that this angioinhibition is dependent on FcγR-mediated signaling. Bevacizumab induced phosphorylation of FcγRI in the mouse cornea (Figure 3b); therefore, we examined the potential involvement of c-Cbl, a major regulator of tyrosine kinase signaling that is downstream of FcγRI-initiated signaling.^73,74^ We found that bevacizumab induced phosphorylation of c-Cbl in wild-type but not FcR NOTAM mouse BMDMs (Figure 6e). This was also corroborated *in vivo*: increased phosphorylation of c-Cbl was observed in the corneas of wild-type mice treated with bevacizumab and bevacizumab-Fc, but not bevacizumab-Fab, following suture injury (Figure 6f). Neither bevacizumab nor human IgG1 inhibited mouse Vegfa-induced migration of *c-Cbl*^−/−^ BMDMs (Figure 6c). Further, we found that bevacizumab did not inhibit corneal or choroidal angiogenesis in *c-Cbl*^−/−^ mice (Figure 6g), indicating that c-Cbl activation is essential for this process.

One of the principal signaling pathways employed by mouse Vegfa to induce macrophage migration is activation of Vegfr1 receptor tyrosine kinase and downstream activation of PI3K and PLCγ1.^75–78^ Via its E3 ubiquitin ligase activity, c-Cbl is capable of inducing degradation of numerous tyrosine kinases including Vegfr1.^79^ Indeed, we found that bevacizumab treatment of mouse macrophages induced degradation of Vegfr1 in wild-type but not FcR NOTAM BMDMs (Figure 6h). Bevacizumab also reduced mouse Vegfa-induced phosphorylation of PI3K and PLCγ1 in mouse macrophages (Figure 6i). Consistent with these findings, neither bevacizumab nor human IgG1 inhibited mouse Vegfa-induced migration of BMDMs isolated from c-Cbl (C379A) mutant mice (Figure 6c), which lack a functional RING finger domain necessary for the E3 ubiquitin ligase activity of c-Cbl.^80^ Also consistent with these findings, and the lack of angioinhibition observed in *c-Cbl*^−/−^ mice, was the finding that bevacizumab did not reduce corneal angiogenesis in c-Cbl (C379A) mutant mice (Supplementary Figure 23). We also found that bevacizumab induced phosphorylation of c-CBL and degradation of VEGFR1 in primary human peripheral blood monocytes as well as in THP-1 human monocytes (Figure 6j).

### Human IgG1 does not inhibit angiogenesis via ADCC, ADCP or CDC

Antibody-dependent cell-mediated cytotoxicity (ADCC) and antibody-dependent cellular phagocytosis (ADCP) are two-step processes initiated by full-length IgGs that couple Fab binding to a target cell antigen with Fc binding to an activating FcγR on an effector cell.^71^ These effector functions, as well as complement-dependent cytotoxicity (CDC) have a major role in the mode of action of several monoclonal antibodies employed in cancer therapy.^81–83^ Our findings that numerous human IgG1 antibodies, each with different Fab targeting domains (and none of which target mouse antigens), similarly suppressed angiogenesis argue against ADCC and ADCP as the mediators of this class effect. Moreover, bevacizumab-Fc and human IgG1-Fc, each devoid of Fab domains, also suppressed angiogenesis like full-length antibodies. We have already shown that bevacizumab inhibited corneal and choroidal angiogenesis in mice lacking FcγRIII, a receptor on NK cells that mediates ADCC,^84^ and in mice lacking FcγRIV, which also has an important role in ADCC^85^ (Supplementary Figures 8 and 9). In addition, bevacizumab inhibited corneal and choroidal angiogenesis in *Il2rg*^*−/−*^ mice, which are deficient in NK cells (Supplementary Figure 24). These data support the thesis that this effector function is not involved in the angioinhibitory effect of bevacizumab in mice.

The inability of denosumab to suppress corneal or choroidal angiogenesis suggests that ADCC and CDC, which can be induced by both human IgG1 and human IgG2,^86^ are not responsible for angioinhibition induced by human IgG1s. We also found that a mutant version of alemtuzumab (G1Δa), which was engineered with point mutations in the CH2 domain of its Fc region that eliminate its CDC activity, yet retains binding to human CD52 and to FcγRI (Supplementary Figure 15a–c), inhibited corneal and choroidal angiogenesis in wild-type mice (Supplementary Figure 15d and e). In addition, bevacizumab suppressed choroidal angiogenesis in *C1qa*^−/−^ mice (Supplementary Figure 25), which are deficient in complement C1QA, confirming that the angioinhibitory activity of human IgG1 antibodies does not require CDC. Subretinal transfection of a plasmid encoding a mutant form of human IgG1-Fc engineered with the K322A or D270A point mutations, which eliminates binding to C1q and induction of CDC while preserving binding to FcγRI,^66,67,87^ also reduced choroidal angiogenesis in wild-type mice (Supplementary Figure 26). Collectively, these data support the concept that angioinhibition is a target-independent class effect of human or humanized IgG1 monoclonal antibodies that is mediated via FcγRI, and not ADCC, ADCP or CDC.

## Discussion

We have shown that human or humanized IgG1 antibodies are, as a class, angioinhibitory in multiple mouse models of ocular and muscle angiogenesis via Fc-dependent signaling. Our findings introduce angiosuppression to the list of important biological functions that are triggered by FcγRI *in vivo*.^57^ Exploiting this intrinsic property of human IgG1s could offer new therapeutic opportunities to treat diseases driven by angiogenesis that collectively affect nearly 10% of the world’s population.^20^ For example, several human IgG1 drugs or human IgG1-Fc fusion proteins approved for other indications could be repurposed as angiogenesis inhibitors. IVIg or non-targeted, ‘generic’ human IgG-Fc might represent even more inexpensive alternatives, as we demonstrate in a companion manuscript.^88^ Additional anti-angiogenic efforts might be directed toward developing peptides or small molecules that induce signaling via FcγRI or c-Cbl.

The dose of bevacizumab (100 μg) we injected into the mouse cornea, whose volume is ~2 μl, is similar in concentration to the dose of bevacizumab (2.5–5 mg) that has been administered into human corneas, whose volume is ~70 μl. Our findings suggest that in human corneas, bevacizumab would, at this dose, exert anti-angiogenic activity both via VEGFA inhibition and via FcγRI-mediated pathways, and that it might be expected to suppress angiogenesis to a greater extent than ranibizumab, which possesses only the anti-VEGFA activity. Indeed, a recent prospective randomized study reported that in humans with corneal angiogenesis, bevacizumab was superior to ranibizumab.^18^

In contrast, no significant difference was found between bevacizumab and ranibizumab in human eyes with choroidal angiogenesis due to neovascular AMD.^4,19^ We suggest that the reason for this lack of difference is that the amount of bevacizumab that is currently administered in these patients, while sufficient to neutralize VEGFA, is insufficient to induce FcγRI-mediated signaling. The dose of intravitreously administered bevacizumab required to suppress choroidal angiogenesis via FcγRI in mice (25 μg) translates, based on relative vitreous humor volumes, to ~10 mg in the human eye, which is eightfold the currently administered clinical dose. These values are compatible with the relative lower affinity of human IgG1-Fc for human FcγRI (*K*_D_=15–40 nmol/l)^45,56,89^ compared with that of bevacizumab for human VEGFA (*K*_D_=0.5–2.2 nmol/l).^3,5^ Our findings predict that a eightfold higher dose bevacizumab would achieve both VEGFA inhibition and FcγRI-mediated angioinhibition, and provide a rationale for testing such higher doses of bevacizumab or combining human IgG1-Fc to anti-human VEGFA drugs in patients with neovascular AMD to potentiate therapeutic angioinhibition.

It is reasonable to query whether it would be possible to inject 10 mg of bevacizumab into the human eye. The viability of injecting 10 mg of a biological drug has been demonstrated in a Phase 2 trial of lampalizumab, a Fab fragment. At present in the clinic, 1.25 mg of bevacizumab is injected in a 50-μl volume. Retina specialists routinely inject 100 μl of corticosteroids or 200 μl of antibiotics into the vitreous humor of humans for various disorders. With such higher delivery volumes, 10 mg of bevacizumab can be administered by increasing the concentration of the formulation from the current 25 mg/ml to 50–100 mg/ml, a value similar to that of therapeutic human IVIg preparations in current use. Alternatively, bevacizumab-Fc or human IgG1-Fc could be administered, at correspondingly lower doses, to induce FcγRI-dependent angioinhibition.

It would be interesting to explore to what extent the therapeutic effects of IgG1 antibodies used in the treatment of AMD, arthritis, asthma and solid tumors—disorders in which angiogenesis plays a critical role^20^—might be mediated by FcγRI. Our data also suggest that it might be prudent to monitor potential effects of human IgG1 antibodies on the vasculature in other diseases, as we demonstrate is the case in IVIg-treated patients in a companion manuscript.^88^ Indeed, the minimal angioinhibitory dose of bevacizumab in mice, 15 mg/kg, is used in humans with many forms of cancer, suggesting that at this dose in people, the drug might have dual anti-angiogenic activity: via VEGFA inhibition and FcγRI-dependent pathways. Although most human IgG1 antibodies are administered systemically at doses of 5–10 mg/kg, several are administered at 15 mg/kg and some as high as 30 mg/kg. Whether these antibodies might modulate other cellular processes, apart from angioinhibition, via FcγRI/c-Cbl signaling also merits future study. Such effects, if they occur, could be mitigated by the use of miniaturized configurations such as Fab or single chain variable fragments, fully deglycosylated antibodies, or Fc region engineering. Prolonged and frequent therapeutic IgG injections could potentially interfere with natural activation of FcγRs by endogenous IgGs. Therefore, targeted local therapy on an intermittent basis might be preferable for the treatment of chronic diseases.

Our bone marrow chimera experiments point to FcγRI on circulating myeloid cells as being critical for bevacizumab-induced angioinhibition. In human AMD as well as murine laser-induced angiogenesis, macrophages are highly spatially and temporally coincident with areas of choroidal neoangiogenesis.^64,90^ Indeed, of the various circulating myeloid cells in mice, only macrophages express FcγRI.^91^ Furthermore, we documented a reduction in macrophage infiltration following bevacizumab treatment that corresponds to angioinhibition. Nevertheless, in addition to disrupting Vegfr1 levels and signaling in macrophages, FcγRI-mediated events might also affect other myeloid cells, endothelial cells, or their bone marrow-derived precursors, and could transduce complex crosstalk among these cell types to modulate angiogenesis. Signaling pathways downstream of c-Cbl activation, as well as other yet to be determined molecular signals triggered via FcγRI, could be additionally responsible for human IgG1-induced angioinhibition.

Our data suggest that endogenous Igs could have a homeostatic role in modulating physiological or pathological angiogenesis. Future studies could explore the extent to which Igs regulate developmental vasculature. Polymorphisms in various *FCGR* genes have been associated with clinical responses to certain monoclonal antibodies in cancer.^92^ It would be interesting to explore whether variants in *FCGR1* might affect the vascular status or clinical response of patients to various human IgG1 antibodies, when they are administered at doses that would be expected to induce FcγRI-mediated signaling.

Our studies, which have identified an unexpected vascular effect of widely used drugs, highlight the importance of employing rigorous biological controls for studies of IgGs. In revealing the intrinsic anti-angiogenic capacity of Fc-containing human IgG1s, these findings could be instructive in the future design and use of antibody-based therapeutics, expand understanding of the biological links between immunity and angiogenesis, and potentially enable novel angioinhibitory therapies.
