# Supplementary material for: Human IgG1 antibodies suppress angiogenesis in a target-independent manner
Source: Signal Transduct Target Ther. 2016 Jan 28;1:15001–. doi: 10.1038/sigtrans.2015.1 (PMC4763941; doi:10.1038/sigtrans.2015.1)
Supplement: Supplementary Figures [file sigtrans20151-s1.pdf]

Figure S1

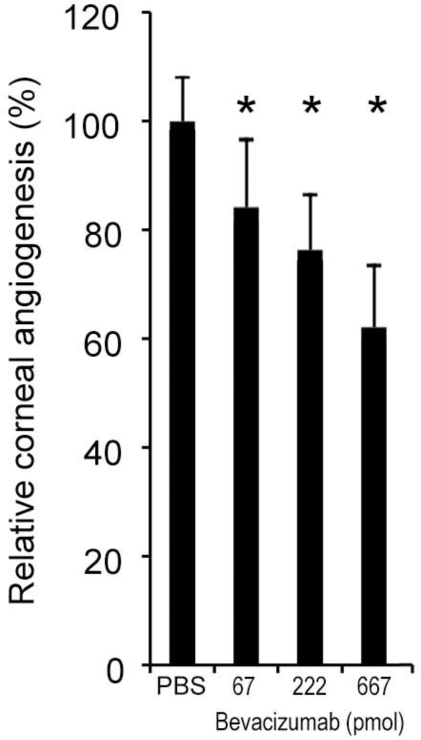

Supplementary Figure 1. Bevacizumab inhibited corneal angiogenesis in a dose-dependent manner. Results are means ± SEM. * $P < 0.05$ compared with PBS.

Figure S2

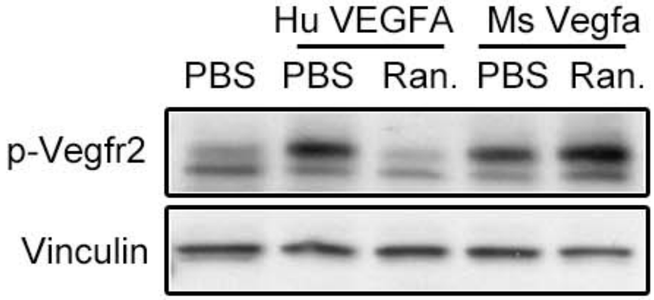

Supplementary Figure 2. Western blot shows that ranibizumab inhibited Vegfr2 phosphorylation (pVegfr2) in Py4 mouse hemangioma endothelial cells when treated with human VEGFA, but not when treated with mouse Vegfa, after 10 minutes. Protein loading was assessed by Vinculin abundance. Image representative of 3 experiments.

Figure S3

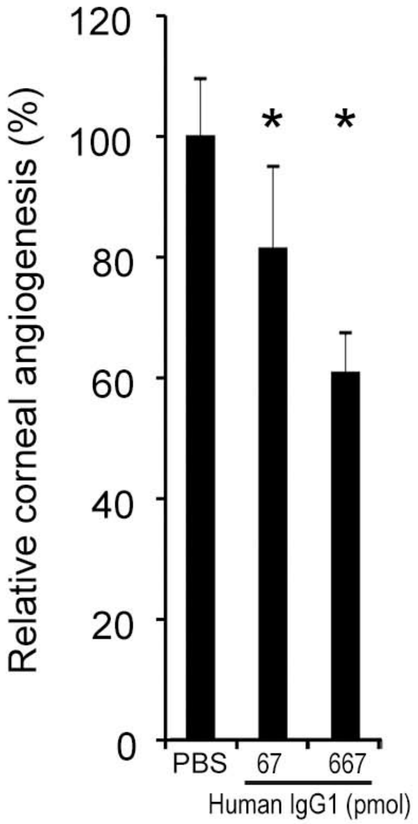

Supplementary Figure 3. Human IgG1 inhibited corneal angiogenesis in a dose-dependent manner. Results are means ± SEM. * $P < 0.05$ compared with PBS.

Figure S4

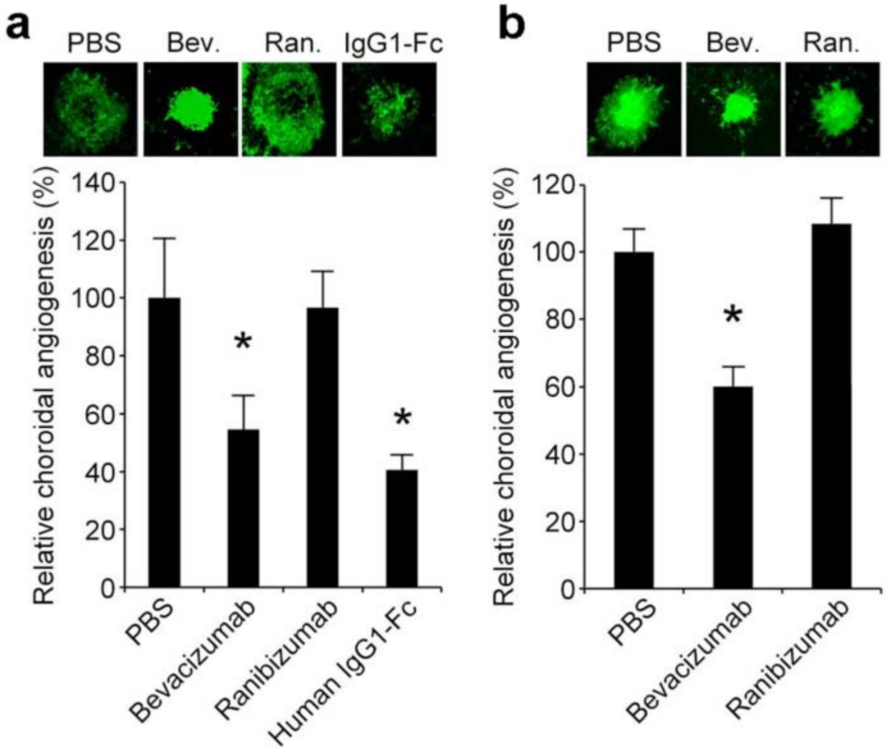

Supplementary Figure 4. (a, b) Bevacizumab, and human IgG1-Fc, but not ranibizumab, suppressed choroidal angiogenesis in wild-type mice 7 days after laser injury compared with PBS (experiments performed in Y.O. **(a)** and H.T. **(b)** laboratories). Images depict representative choroidal angiogenesis lesions in each treatment group. Results are means ± SEM (n = 8–10). * $P < 0.05$ compared with PBS.

Figure S5

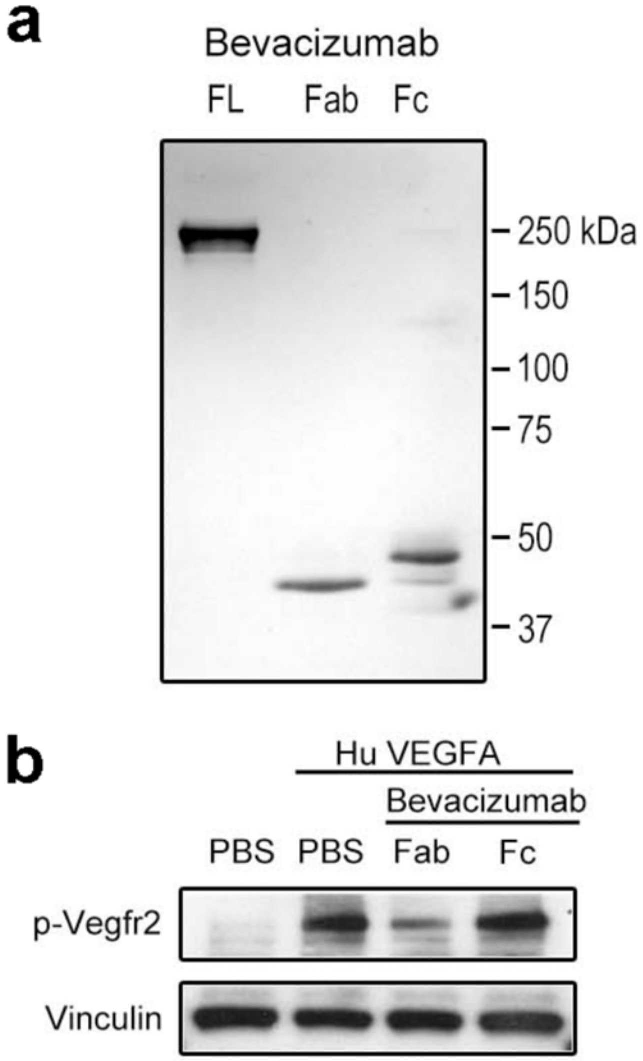

Supplementary Figure 5. (a) Coomassie stained SDS-page gel with complete separation of Fab and Fc fragments of full-length (FL) bevacizumab after papain digestion. **(b)** Western blot shows that the Fab fragment but not the Fc fragment of bevacizumab inhibited Vegfr2 phosphorylation (pVegfr2) in Py4 mouse hemangioma endothelial cells when treated with mouse Vegfa after 10 minutes. Protein loading was assessed by Vinculin abundance. Image representative of 3 experiments.

Figure S6

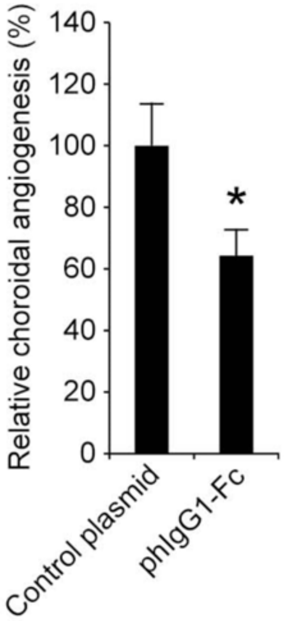

Supplementary Figure 6. *In vivo* subretinal transfection of a plasmid encoding human IgG1-Fc (phIgG1-Fc) reduced choroidal angiogenesis in wild-type mice. Results are means ± SEM (n = 10–12). * $P < 0.05$ compared with control plasmid.

Figure S7

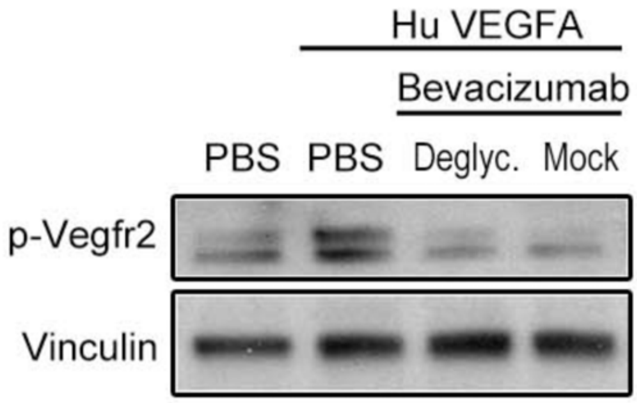

Supplementary Figure 7. Western blot shows that both deglycosylated bevacizumab and mock-treated bevacizumab inhibited Vegfr2 phosphorylation (pVegfr2) in Py4 mouse hemangioma endothelial cells when treated with human VEGFA after 10 minutes. Protein loading was assessed by Vinculin abundance. Image representative of 3 experiments.

Figure S8

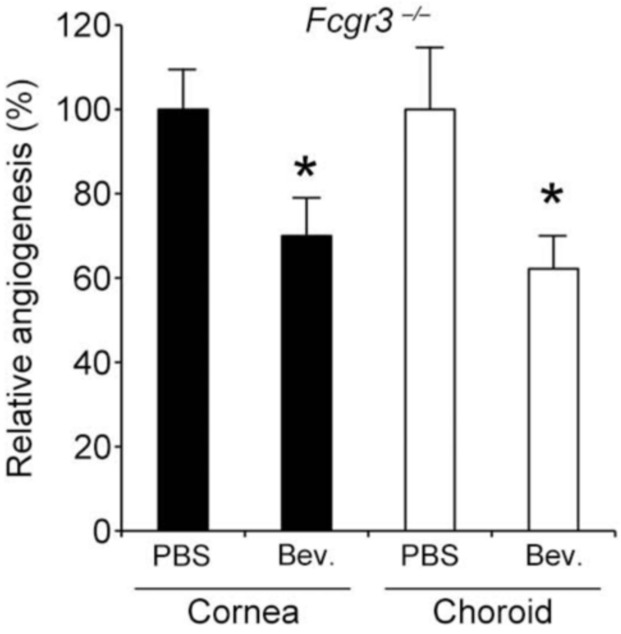

Supplementary Figure 8. Bevacizumab (Bev.) inhibited corneal and choroidal angiogenesis in *Fcgr3*$^{-/-}$ mice (which lack FcγRIII). Results are means ± SEM (n = 13–16). * $P < 0.05$ compared with PBS.

Figure S9

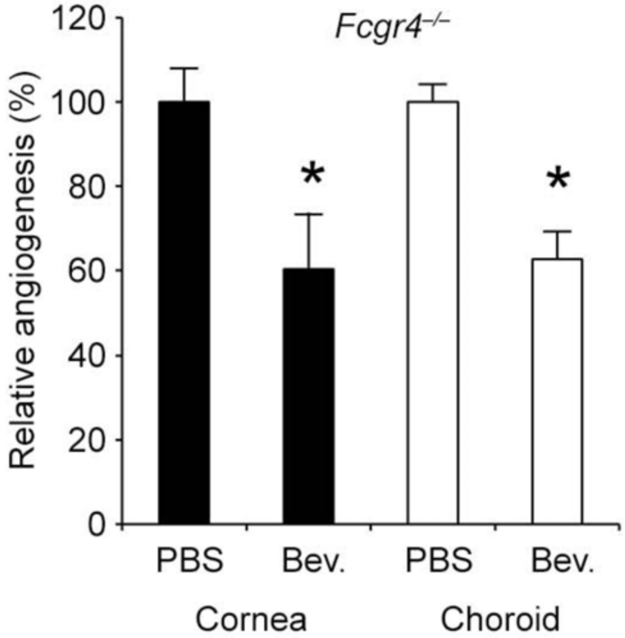

Supplementary Figure 9. Bevacizumab (Bev.) inhibited corneal and choroidal angiogenesis in *Fcgr4*$^{-/-}$ mice (which lack FcγRIV). Results are means ± SEM (n = 6). * $P < 0.05$ compared with PBS.

Figure S10

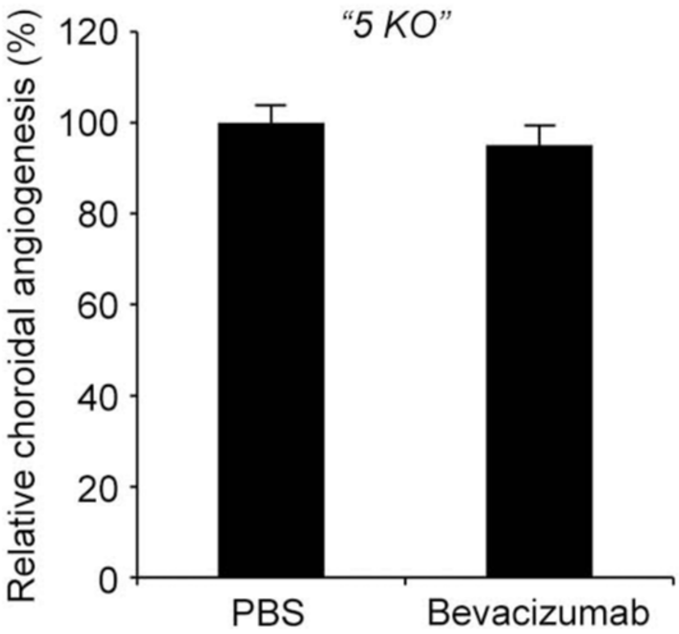

Supplementary Figure 10. Bevacizumab did not inhibit choroidal angiogenesis in *Fcgr1*$^{-/-}$; *Fcgr2b*$^{-/-}$; *Fcgr3a*$^{-/-}$; *Fcer1a*$^{-/-}$; *Fcer2a*$^{-/-}$ mice ("5KO" mice that express FcγRIV but not any other IgG or IgE receptors). Results are means ± SEM (n = 6). * $P < 0.05$ compared with PBS.

Figure S11

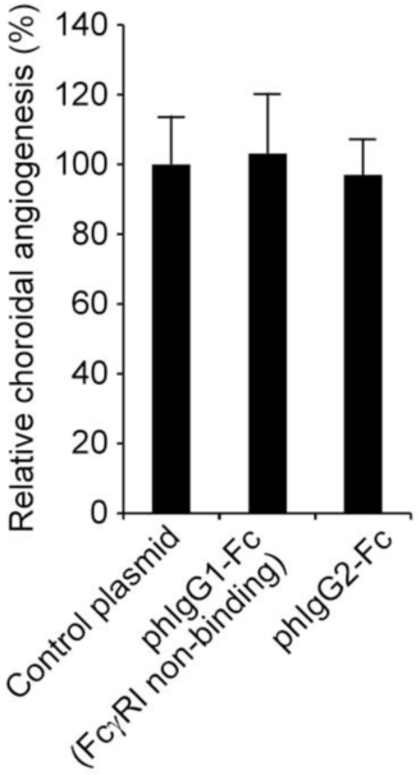

Supplementary Figure 11. *In vivo* subretinal transfection of a plasmid encoding a mutant form of human IgG1-Fc that does not bind FcγRI (pFUSE-hIgG1e3-Fc2) or of a plasmid encoding human IgG2-Fc did not suppress choroidal angiogenesis in wild-type mice. Results are means ± SEM (n = 10–12). No significant differences from control plasmid.

Figure S12

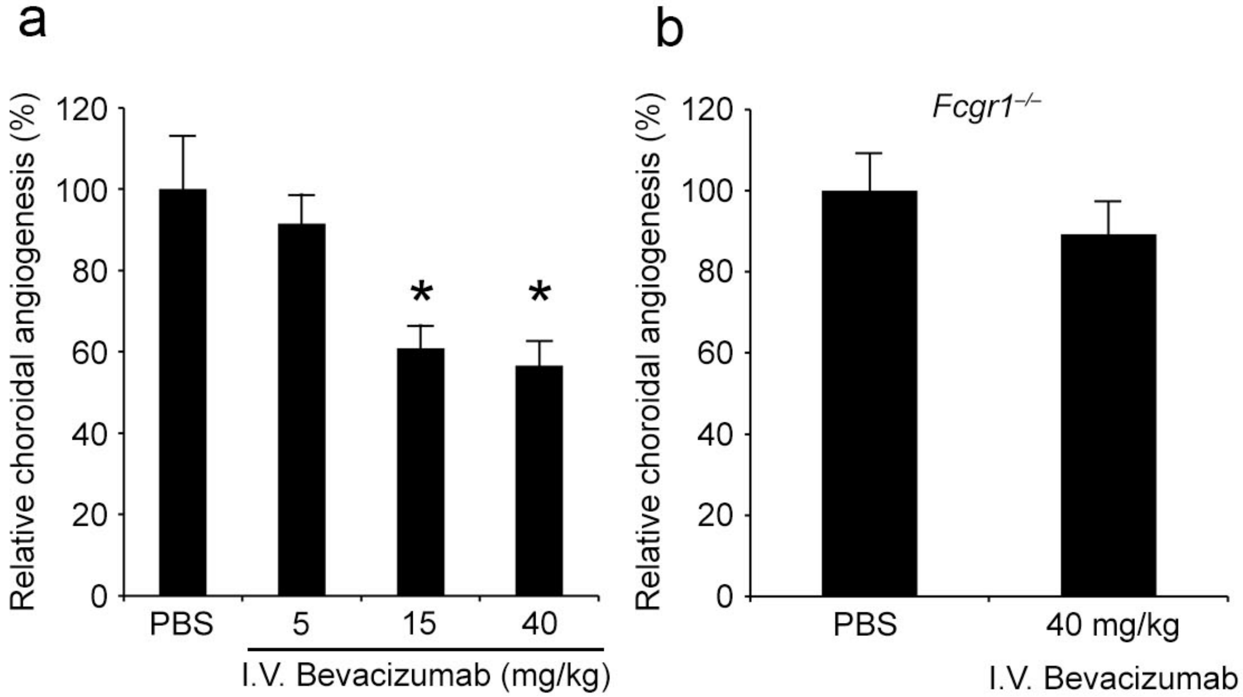

Supplementary Figure 12. (a, b) Intravenous administration of bevacizumab inhibited choroidal angiogenesis in wild-type mice in a dose-dependent manner **(a)** but did not do so in *Fcgr1*$^{-/-}$ mice **(b)**. Results are means ± SEM (n = 10–12). * $P < 0.05$ compared with PBS **(a)**. No significant difference **(b)**.

Figure S13

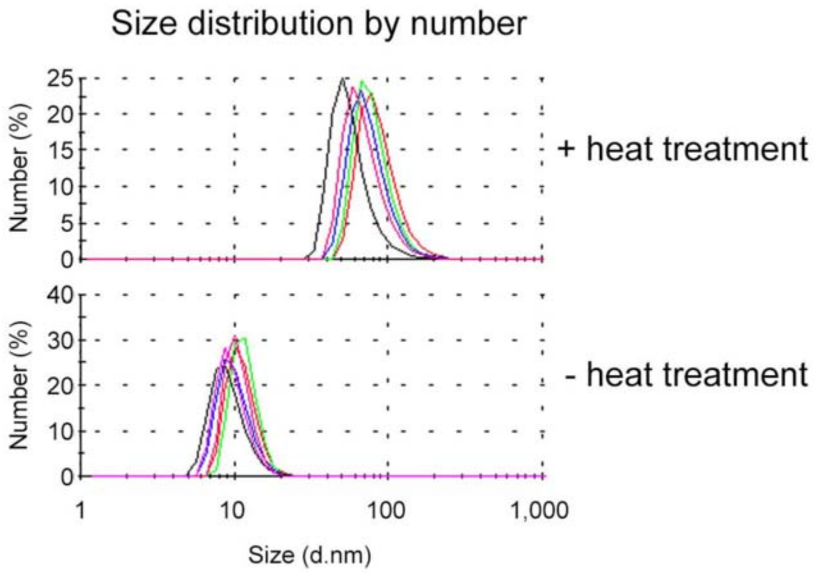

Supplementary Figure 13. Size distribution profiles obtained by dynamic light scattering for bevacizumab show an average diameter of ~ 10 nm before (top) and ~ 70 nm after (bottom) heat-induced aggregation. n = 5–6.

Figure S14

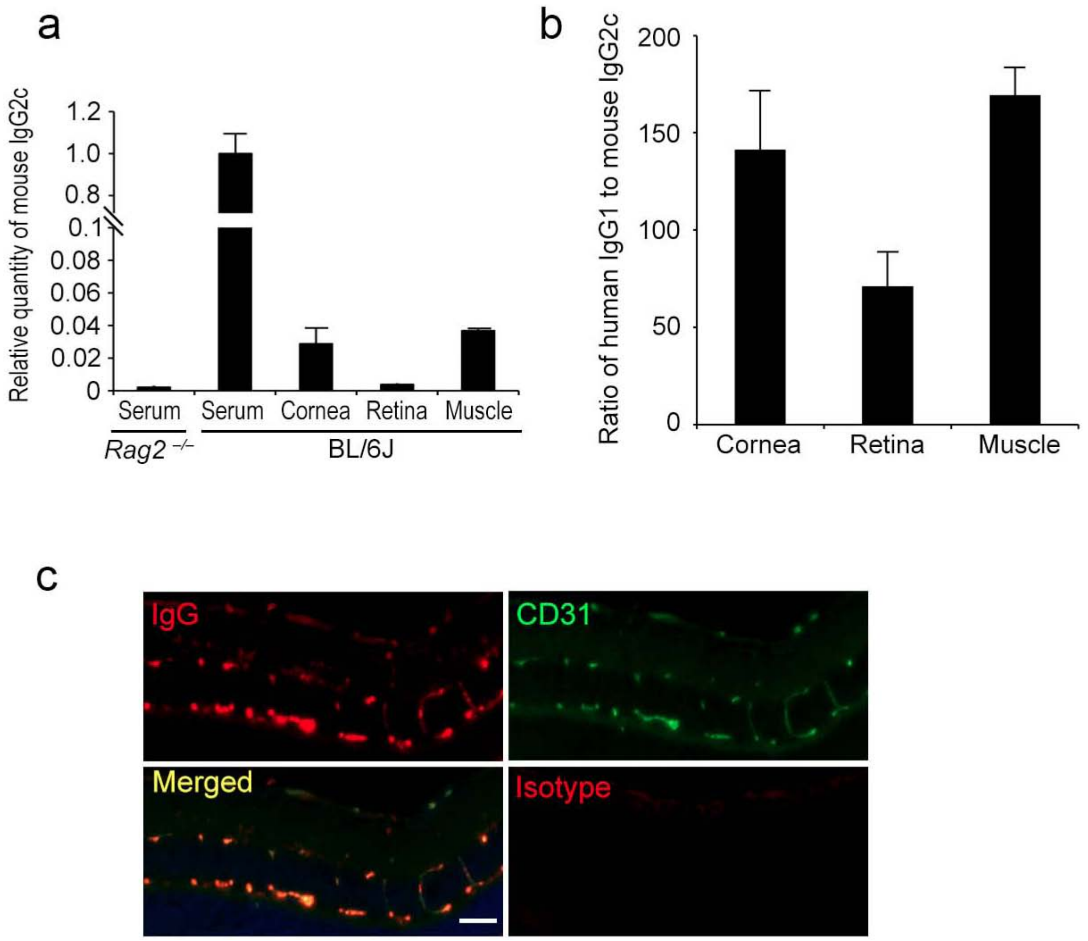

Supplementary Figure 14. (a) Enzyme-Linked Immunosorbent Assay (ELISA) shows that the levels of endogenous mouse IgG2c in wild-type mouse corneas, retinae, or hind limb muscles (following whole body perfusion to remove blood), one day after injury, are a very small fraction of serum IgG2c levels, approaching the levels observed in the serum of *Rag2*$^{-/-}$ mice, which lack B and T lymphocytes. . Levels normalized to mean serum IgG2c levels in C57BL/6J mice. **(b)** ELISA shows that in wild-type mouse corneas, retinae, or hind limb muscles treated with bevacizumab, levels of exogenous human IgG1 (following whole body perfusion to remove blood), at one day after injection, are much higher than levels of endogenous mouse IgG2c. n = 6–7 **(a, b)**. **(c)** Representative images of wild-type mouse retina shows immunolocalization of endogenous mouse IgG (red) that is abundant and spatially confined to the

retinal blood vessels (CD31, green), and nearly undetectable in the extravascular tissue. Representative image of wild-type mouse retina probed with isotype control antibody shows specificity of IgG staining. Nuclei were stained with DAPI. Scale bar, 50 µm. (n = 3).

Figure S15

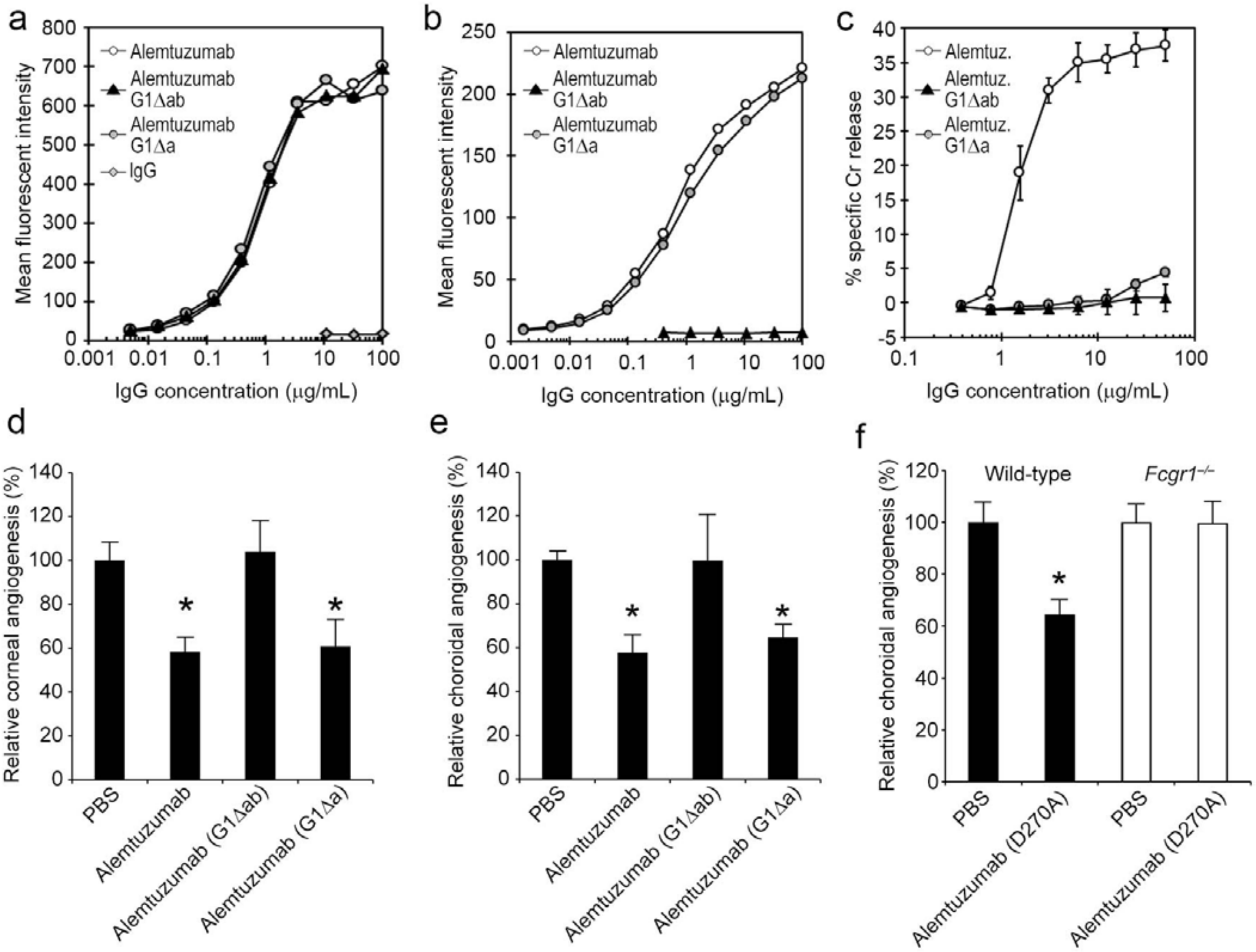

Supplementary Figure 15. (a) Alemtuzumab IgG1 and mutant alemtuzumab G1Δab and G1Δa antibodies were equivalent in antigen-binding as observed by the detection of near-identical binding to CD52 on the surface of human lymphocytes. Binding to Fc receptors was blocked by the presence of normal rabbit serum as indicated by the lack of binding of a control human IgG1 antibody (IgG) with irrelevant specificity. **(b)** The antibodies were compared in their ability to interact with FcγRI on the surface of a transfected cell line in a monomeric IgG binding assay. Alemtuzumab IgG1 and alemtuzumab G1Δa antibodies bound strongly to human FcγRI but the modifications of mutant alemtuzumab G1Δab reduced this binding by at least 10^4-fold. **(c)** Alemtuzumab, but not

mutant alemtuzumab G1Δab and G1Δa antibodies, induced complement-mediated lysis, measured as % specific ^{51}Cr release from human PBMC in the presence of human serum (mean ± SD of triplicate samples). **(a–c)** Each graph representative of 3 experiments. **(d, e)** Inhibition of angiogenesis requires binding to FcγRI but not to CD52. Alemtuzumab and alemtuzumab G1Δa, but not alemtuzumab G1Δab, inhibited corneal (**d**) and (**e**) choroidal angiogenesis in wild-type mice compared to PBS. (**f**) Alemtuzumab containing the D270A mutation, which preserves binding to FcγRI but loses binding to FcγRII & FcγRIII and does not induce complement dependent cytotoxicity, inhibited choroidal angiogenesis in wild-type mice but not in *Fcgr1*$^{-/-}$ mice. Results are means ± SEM (n = 8–30). * $P < 0.05$ compared with PBS **(d–f)**.

Figure S16

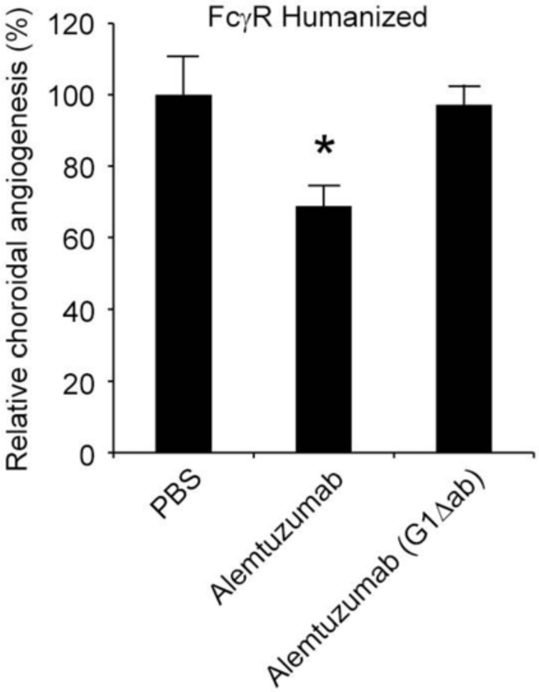

Supplementary Figure 16. Alemtuzumab inhibited choroidal angiogenesis in FcγR humanized mice compared to PBS. In contrast, mutant alemtuzumab G1Δab, which was engineered with point mutations in the CH2 domain of its Fc region that eliminate binding to FcγRI and reduce binding to other FcγRs, did not suppress angiogenesis in these mice. Results are means ± SEM (n = 8–30). * $P < 0.05$ compared with PBS.

Figure S17

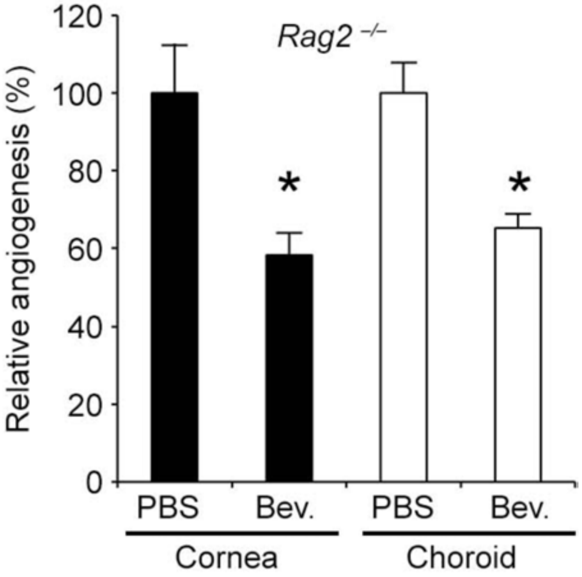

Supplementary Figure 17. Bevacizumab (Bev.) inhibits corneal and choroidal angiogenesis in *Rag2*$^{-/-}$ mice, which lack B and T cells. Results are means ± SEM (n = 8–10, cornea; n = 6, choroid). * $P < 0.05$ compared with PBS.

Figure S18

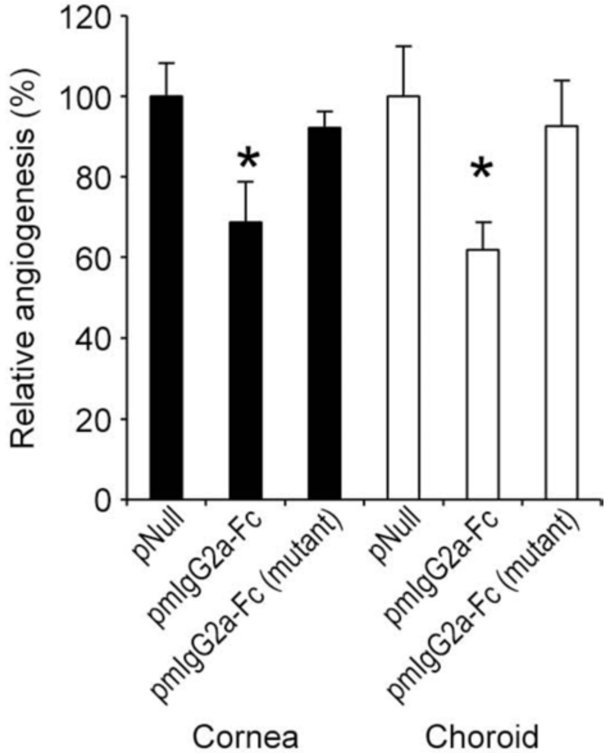

Supplementary Figure 18. *In vivo* transfection of a plasmid encoding mouse IgG2a-Fc (pmIgG2a-Fc) inhibits corneal and choroidal angiogenesis in wild-type mice, compared to a control pNull plasmid. A mutant pmIgG2a-Fc engineered with multiple point mutations that reduce binding to FcγRI (pFUSE-mIgG2Ae1-Fc2) did not inhibit angiogenesis. Results are means ± SEM (n = 6–7). * $P < 0.05$ compared with pNull.

Figure S19

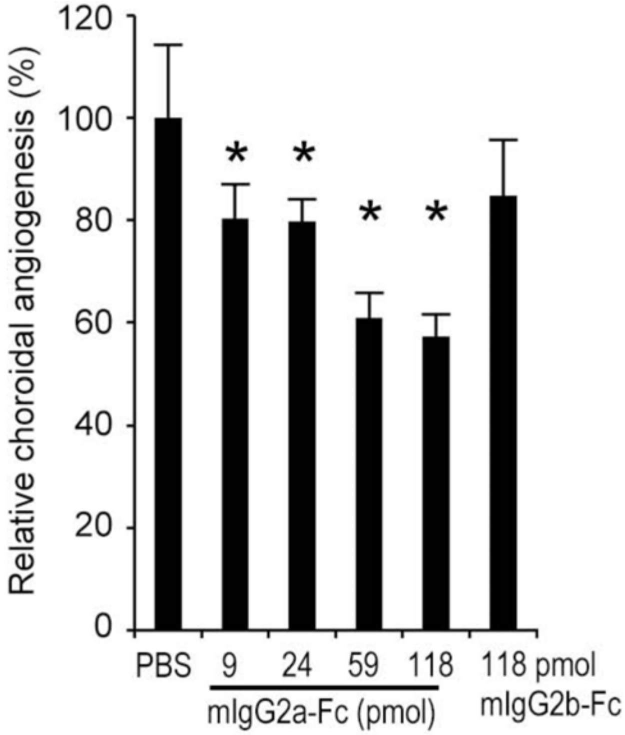

Supplementary Figure 19. Intravitreous administration of recombinant mouse IgG2a-Fc, but not of mouse IgG2b-Fc, which does not bind FcγRI, inhibits choroidal angiogenesis in wild-type mice, compared to PBS. Injected amounts listed as picomoles (pmol). Results are means ± SEM (n = 6–8). * $P < 0.05$ compared with PBS.

Figure S20

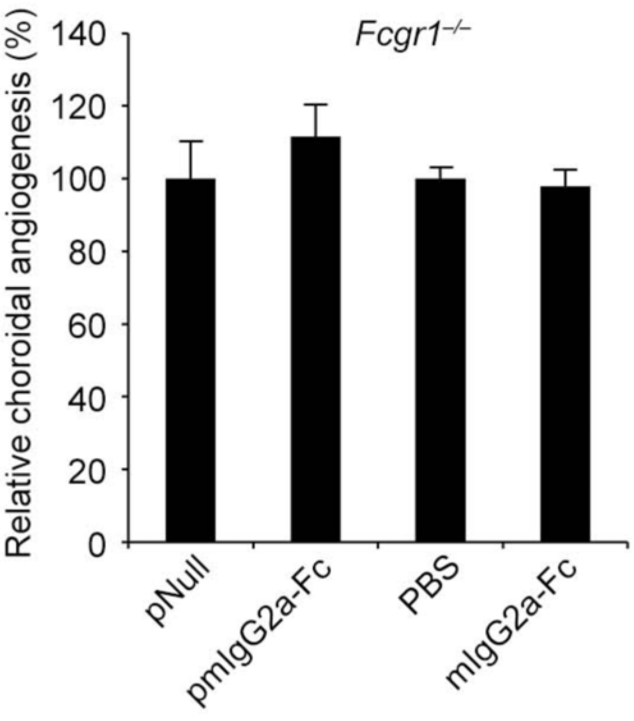

Supplementary Figure 20. Neither *in vivo* subretinal transfection of a plasmid encoding mouse IgG2a-Fc (pmIgG2a-Fc) nor intravitreous administration of recombinant mouse IgG2a-Fc (118 picomoles) inhibited choroidal angiogenesis in *Fcgr1*$^{-/-}$ mice. Results are means ± SEM (n = 6). No significant differences compared with pNull or PBS.

Figure S21

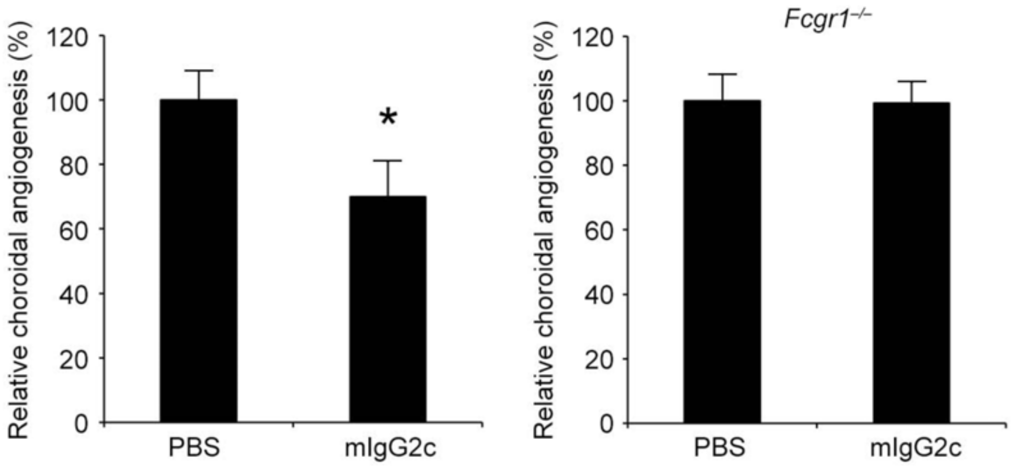

Supplementary Figure 21. Intravitreous administration of purified mouse IgG2c (167 picomoles) inhibits choroidal angiogenesis in wild-type mice (left panel) but not in *Fcgr1*$^{-/-}$ mice (right panel), compared to PBS. Results are means ± SEM (n = 6). * $P < 0.05$ compared with PBS.

Figure S22

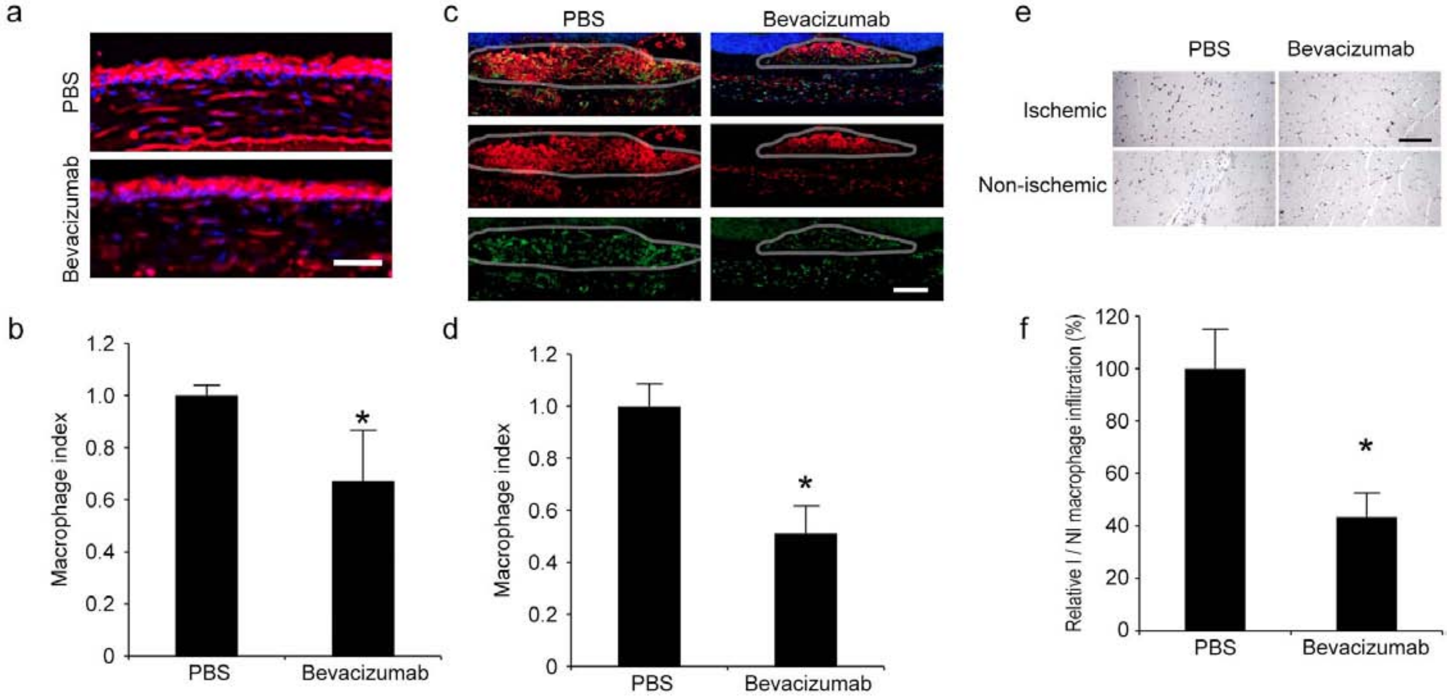

Supplementary Figure 22. Reduced number of F4/80+ (red) macrophages in bevacizumab treated corneas, as compared with PBS treatment, as seen in **(a)** representative pictures and **(b)** macrophage quantification, 3 days after suture injury. Autofluorescence of corneal epithelium is seen in both PBS and bevacizumab treated corneas. **(c, d)** Reduced number of F4/80+ (red), CD45+ (green) double-positive macrophages in choroidal neovascular lesions of eyes treated with bevacizumab, as compared with PBS treatment, as seen in **(c)** representative pictures and **(d)** macrophage quantification, 3 days after laser injury. **(e, f)** Reduced number of F4/80+ (brown) macrophages in muscle of ischemic hind limbs treated with bevacizumab, as compared with PBS treatment, as seen in **(e)** representative pictures **(f)** and macrophage quantification, 4 days after surgery. Scale bars, 50 µm (**a**), 100 µm (**c, e**). Results are means ± SEM (n = 6–8). * $P < 0.05$ compared with PBS.

Figure S23

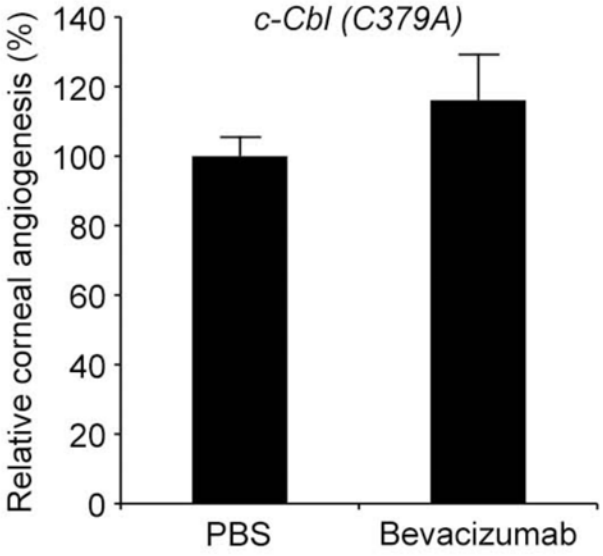

Supplementary Figure 23. Bevacizumab did not reduce corneal angiogenesis in c-Cbl (C379A) mutant mice, which lack a functional RING finger domain necessary for the E3 ubiquitin ligase activity of c-Cbl. Results are means ± SEM (n = 6). No significant difference compared with PBS.

Figure S24

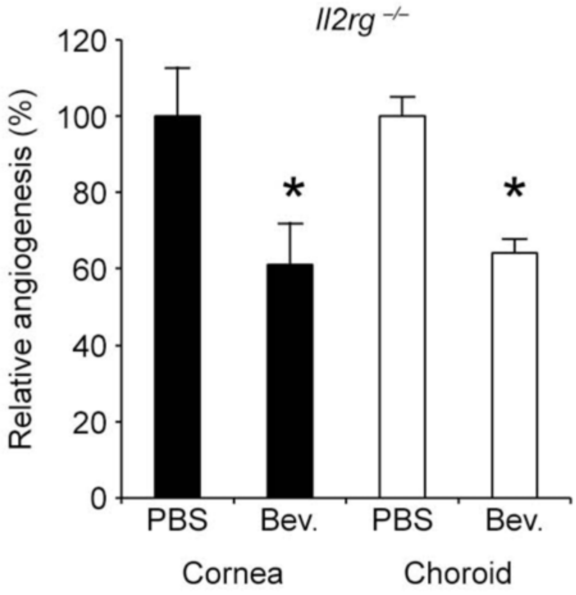

Supplementary Figure 24. Bevacizumab (Bev.) inhibited corneal and choroidal angiogenesis in *Il2rg*$^{-/-}$ mice, which are deficient in NK cells. Results are means ± SEM (n = 13–16). * $P < 0.05$ compared with PBS.

Figure S25

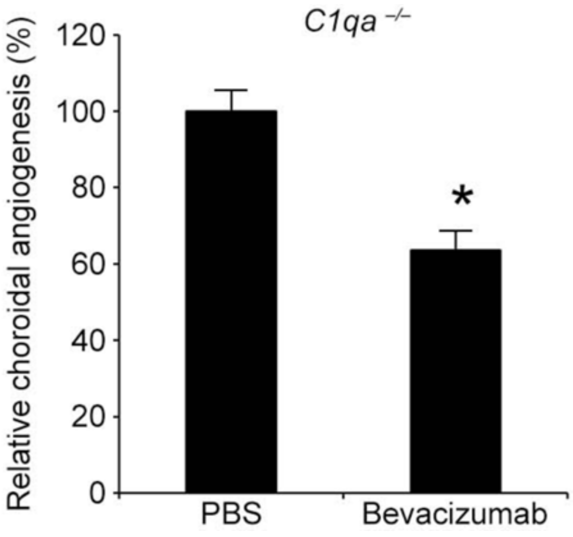

Supplementary Figure 25. Bevacizumab inhibited choroidal angiogenesis in *C1qa*$^{-/-}$ mice. Results are means ± SEM (n = 6). * $P < 0.05$ compared with PBS.

Figure S26

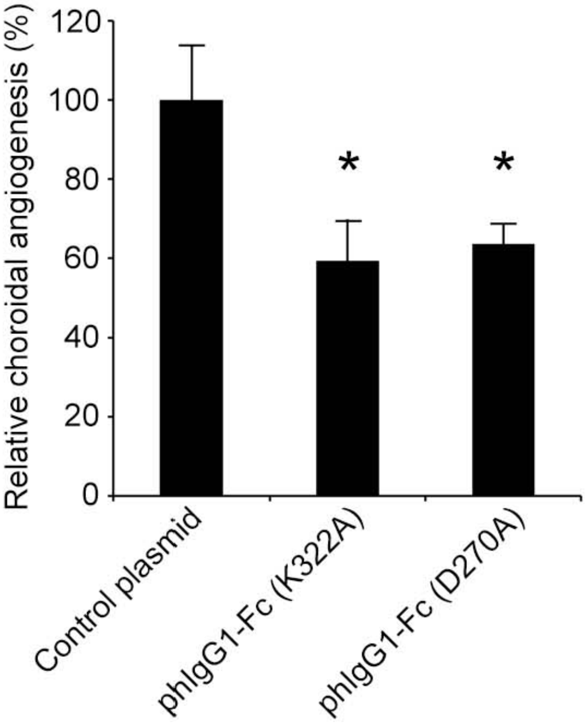

Supplementary Figure 26. *In vivo* subretinal transfection of a plasmid encoding mutant forms of human IgG1-Fc that does not bind C1q or induce CDC (phIgG1-Fc K322A and phIgG1-Fc D270A), reduced choroidal angiogenesis in wild-type mice. Results are means ± SEM (n = 10–12). * $P < 0.05$ compared with control plasmid.

Materials and methods

Animals. All animal experiments were in accordance with the guidelines of the University of Kentucky Institutional Animal Care and Use Committee, and the Association for Research in Vision and Ophthalmology (ARVO) Animal Statement for the Use of Animals in Ophthalmic and Vision Research. The hind limb ischemia experimental procedures were in accordance with European Directives no. 86/609 and Italian D.L. 116 and approved by the veterinarian of the Institute of Genetics and Biophysics. C57BL/6J, *Fcgr3*$^{-/-}$, *Il2rg*$^{-/-}$, *Rag2*$^{-/-}$, and *Tnf*$^{-/-}$ mice were purchased from The Jackson Laboratory (Bar Harbor, ME). *C1qa*$^{-/-}$ (ref. 1), *CD20*$^{-/-}$ (a.k.a. *Ms4a1*$^{-/-}$)[2], *CD52*$^{-/-}$ (ref. 3), *c-Cbl*$^{-/-}$ (ref. 4), *c-Cbl* (C379A)[5], FcγR humanized[6], IgE-deficient[7], *Fcgr1*$^{-/-}$ (ref. 8), *Fcgr4*$^{-/-}$ (ref. 9), *Fcgr1*$^{-/-}$*; Fcgr2b*$^{-/-}$*; Fcgr3*$^{-/-}$*; Fcer1a*$^{-/-}$*; Fcer2a*$^{-/-}$ (ref. 10), FcR NOTAM & *Fcer1g*$^{-/-}$ (ref. 11), and humanized VEGFA mice[12] were obtained from M. Botto & L. Nagy, M.S. Neuberger, M. Okabe, M. Naramura & W.Y. Langdon, J.V. Ravetch, H.C. Oettgen, J.S. Verbeek, P. Bruhns, J.H.W. Leusen, and N. Ferrara respectively. Male mice, aged 4–6 weeks, were randomized 1:1 to treatment with active drug versus inactive drug or control treatments. For all procedures, anesthesia was performed by intraperitoneal injection of 100 mg/kg ketamine hydrochloride (Ft. Dodge Animal Health, Overland Park, KS) and 10 mg/kg xylazine (Phoenix Scientific, St. Joseph, MO). Pupils were dilated with topical tropicamide (1%; Alcon Laboratories, Ft. Worth, TX). Operators were masked to treatment groups while conducting analyses.

Drug injections. Bevacizumab (Avastin™, Roche/Genentech), ranibizumab (Lucentis™, Roche/ Genentech), adalimumab (Humira™, Abbott, Abbott Park, IL), alemtuzumab (Campath™,

Sanofi-Aventis/Genzyme, Cambridge, MA), ofatumumab (Arzerra™, GlaxoSmithKline, Brentford, England), omalizumab (Xolair™, Roche/Genentech), palivizumab (Synagis™, MedImmune, Gaithersburg, MD), tocilizumab (Actemra™, Hoffman-La Roche, Basel, Switzerland), denosumab (Prolia™, Amgen, Thousand Oaks, CA), SU1498 (EMD Millipore, Billerica, MA), human IgG1 (Sigma-Aldrich, St. Louis, MO), human IgG1-Fc (R&D Systems, Minneapolis, MD), mouse IgG2a-Fc or mouse IgG2b-Fc (Sino Biological, Daxing, China), and mouse IgG2c (SouthernBiotech, Birmingham, AL) were used. In corneal angiogenesis experiments, they were injected (0.067–0.667 nmol) into corneal stroma of mice using a 33-gauge Exmire Microsyringe (Ito Corporation) on days 0 (immediately after suture placement), 4, and 8 following suture injury. In choroidal angiogenesis experiments, these drugs were injected (0.009–0.167 nmol) into the vitreous humor of mice using a 33-gauge double-caliber needle (Ito Corporation, Tokyo, Japan) once, immediately after laser injury as previously described. In hind limb angiogenesis experiments, they were intramuscularly injected (2.5 nmol) into each hind limb on days 0 (immediately after surgery) and 2 following surgery. PAM peptide (IgG Fc antagonist) or control peptide were intravitreously (0.1 mg) or intramuscularly (1 mg) administered. Cholesterol-conjugated 17+2-nt siRNAs targeting human *FCGR1A* (GAGAAACAGACAGAAAGdTdT) or firefly *Luc* (UAAGGCUAUGAAGAGAUdTdT) were intravitreously (2 μg) administered. Human VEGFA (0.357 pmol; R&D Systems) was injected intravitreously 1 day before laser injury.

Bevacizumab deglycosylation. Bevacizumab was deglycosylated using Protein Deglycosylation Mix (New England Biolabs, Ipswich, MA) under non-denaturing reaction conditions following the manufacturer’s instructions. Mock-treated or deglycosylated bevacizumab were subjected to

SDS-PAGE and Coomassie staining to assess mobility shift.

Bevacizumab Fab/Fc fragmentation. Bevacizumab Fc and Fab fragments were prepared by using a ImmunoPure Fab Preparation Kit (Thermo Fisher Scientific, Waltham, MA) according to the manufacturer's instructions. Briefly, 4 mg of bevacizumab was mixed with 0.5 ml of immobilized papain. The mixture was incubated by shaking overnight at 37 °C. Crude digest was separated from immobilized papain and applied to a protein A column (AffinityPak; Thermo Fisher Scientific). Fab fragments were recovered in the flow through. Fc fragments and undigested IgG bound to the column were eluted with elution buffer. The fragmentation was confirmed in a reducing 4–12% NuPAGE gels stained with SimplyBlue SafeStain (Life Technologies, Carlsbad, CA). The more clear Fab or Fc fraction was chosen and concentrated using a Vivaspin 20 centrifugal concentrator (10 kDa molecular weight cutoff; Sartorius Stedim Biotech, Bohemia, NY).

***In vivo transfection* and site directed mutagenesis of plasmids.** Subretinal injections in mice were performed using a 37-gauge Exmire microsyringe (Ito Corporation)[13]. *In vivo* transfection of plasmids was achieved using 10% Neuroporter (Genlantis, San Diego, CA) for subretinal injection. The pFUSE-hIgG1-Fc2, pFUSE-hIgG2-Fc2, pFUSE-mIgG2a-Fc2, and pFUSE-mIgG2b-Fc2 vectors (Invivogen, San Diego, CA), which encode Fc-fusion proteins expressing the Fc region (CH2 and CH3 domains) of the human IgG1, human IgG2, mouse IgG2a, and mouse IgG2b heavy chains, respectively, and the hinge region, and contain the IL2 signal sequence to enable secretion, as well as pFUSE-hIgG1e3-Fc2 and pFUSE-mIgG2ae1-Fc2 (Invivogen), which contain point mutations that reduce binding affinity for FcγRI, were injected

into the subretinal space or into the corneal stroma. Using a site directed mutagenesis kit (Agilent Technologies, Santa Clara, CA), we engineered, and sequence-verified, the K322A point mutation into pFUSE-hIgG1-Fc2 to eliminate binding to C1q. A null control plasmid was created by digesting pFUSE-hIgG1-Fc2 with ApoI and religating the plasmid backbone to create an empty vector without the hIgG or mouse IgG coding sequences.

Mutant alemtuzumab creation and binding. Mutant forms of alemtuzumab (G1Δab and G1Δa) that have identical, humanized, CD52-specific variable domains to alemtuzumab[14] were created as previously described[15]. Alemtuzumab G1Δab was created by substituting the corresponding IgG2 or IgG4 residues at key positions in the CH2 domain (E233P, L234V, L235A, G236 deleted, A327G, A330S, P331S) to reduce its capacity to mediate effector functions. Alemtuzumab G1Δa contained only the substitutions from IgG4 (A327G, A330S, P331S). The point mutant alemtuzumab D270A was similarly created. For measurement of binding to CD52, human PBMC were obtained from whole blood by density gradient centrifugation and incubated in wash buffer (PBS, 0.1% BSA, 0.1% NaN_3) containing 20% normal rabbit serum for 45 min on ice to block FcγR binding sites. The cells were distributed to wells of a U-bottom 96-well plate, pelleted by centrifugation and re-suspended in samples of the test antibodies in wash buffer + 10% rabbit serum. After 30 min on ice, the cells were washed three times and similarly incubated with 30 μg/ml FITC-conjugated goat anti-human IgG Fab fragments (Jackson ImmunoResearch, Newmarket, United Kingdom). Washed and fixed cells were analyzed using a CyAn ADP flow cytometer and Summit v4.3 software (DakoCytomation, Ely, United Kingdom). Lymphocytes, the vast majority of which are CD52 positive, were selected using the forward and side scatter parameters and the mean fluorescence of at least

15,000 lymphocytes/sample determined. Binding of alemtuzumab antibodies to B2KA cells expressing human FcγRI was measured as previously described[15]. Ability of alemtuzumab antibodies to induce complement-mediated lysis was measured as the specific ^{51}Cr release from labeled human PBMCs during 1 h incubation with antibody and 25% human serum.

Peptide synthesis. The tetrameric tripeptide PAM (D-Arg-D-Thr-D-Tyr)$_4$-L-Lys$_2$-L-Lys-Gly , and the scrambled control peptide (D-Thr-D-Tyr-D-Arg)$_4$-L-Lys$_2$-L-Lys-Gly, (MW 2144 Da), were produced by solid-phase peptide synthesis by using amino acids in the D configuration[16]. Purified peptides were dissolved at the working concentration in PBS.

Corneal angiogenesis. Two interrupted 11–0 nylon sutures (Mani, Utsunomiya, Japan) were placed into the corneal stroma, midway between the central corneal apex and the limbus (approximately 1.25 mm from the limbus), of both eyes of mice as previously described[17,18]. On day 10 after injury, we calculated the mean percentage CD31$^+$Lyve–1$^-$ blood vessel areas for corneal flat mounts with ImageJ (US National Institutes of Health, Bethesda, MD) as previously reported[17,18]. Eyes were excluded in masked fashion from analyses (2% incidence) if anterior chamber hemorrhage suture loosening, or cataract formation occurred at any time during the study period.

Choroidal angiogenesis. Laser photocoagulation (OcuLight GL, IRIDEX, Mountain View, CA) was performed on both eyes of mice to induce CNV as previously described[19,20]. Choroidal angiogenesis volumes were measured by scanning laser confocal microscopy (TCS SP5, Leica, Solms, Germany) 7 days after injury as previously reported with 0.5% FITC-conjugated Isolectin

B4 (Vector Laboratories, Burlingame, CA)[19,20]. Laser lesions were excluded in masked fashion from analyses (5% incidence) if laser photocoagulation did not induce a bubble, if it induced hemorrhage, or if lesions became confluent with one another.

Hind limb ischemia angiogenesis. Mice were anesthetized before undergoing unilateral proximal femoral artery ligation. The right femoral artery was gently isolated, ligated and excised distal to the deep femoral artery and 0.5 cm proximal to the bifurcation in saphenous and popliteal arteries, as previously described[21]. The non-ischemic left limb underwent sham surgery without arterial ligation. On day 7 after surgery, both anterior and posterior muscles from ischemic and non-ischemic hind limbs were harvested and processed for immunohistochemical analysis for vessel quantification. Animals were excluded in masked fashion from analyses (0% incidence) if hemorrhagic death from improper ligation occurred.

Immunostaining of mouse tissue. For staining of eye tissues, eyes were dissected 3 days after injury, cryoprotected in 30% sucrose, embedded in optimal cutting temperature compound (Tissue-Tek OCT; Sakura Finetek, Torrance, CA), and cryosectioned into 10 μm sections. For mouse IgG, immunofluorescent staining was performed with anti-mouse IgG (1:5000; Sigma). For the negative control staining, the primary antibody was omitted. For macrophage staining in the cornea or choroid, immunofluorescent staining was performed with rat antibody against F4/80 (1:50; AbD Serotec, Raleigh, NC) and, for CNV, rabbit antibody against CD45 (1:100; Abcam, Cambridge, MA). Isotype IgG was substituted for the primary antibody to assess the specificity of the staining. Bound antibody was detected with Alexa fluor-conjugated secondary antibodies. Sections were mounted with ProLong Gold Antifade reagent with DAPI

(Life Technologies). In the cornea, a region of 600 μm starting at the limbus and including the whole width of the cornea was delineated, and positive cells within that area were counted. In the choroid, the area within and around the injury site that stained with F4/80 and CD45 antibodies was quantified (Adobe Photoshop, CS6.0; Adobe Systems, San Jose, CA) to obtain a quantitative index of macrophages, as described previously[22-24]. Microglial cells, defined as those expressing little or no CD45, were excluded. For macrophage quantification in muscle, 5 μm-thick muscle cryosections were incubated overnight at 4 °C with the rat anti-mouse F4/80 (1:50). The staining procedure was continued using specific secondary biotinylated antibody (Dako, Glostrup, Denmark). Slides were counterstained with hematoxylin. Images were recorded with a digital camera Leica DC480 (Milano). Densitometry analysis for F4/80 staining was performed with QwinPro software (Leica).

Color laser Doppler analysis. Color laser Doppler analysis was performed 7 days after femoral artery ligation using a dedicated Laser Doppler Perfusion Imaging System (LDPI, PeriScan PIM II System, Perimed AB, Järfälla, Sweden) with high resolution, in single mode. Hind limbs were depilated and mice were placed on a heating plate at 37 °C. The distance between the scanner head and tissue surface was 8 cm. An area of 5 × 5 cm was sequentially scanned and blood flow 1 mm under the surface was measured. Color-coded images were recorded, and analyses were performed calculating the average perfusion of the right and left distal limb. Dark blue color implied low or absent perfusion whereas red implied maximal perfusion.

Macrophage migration assay. Freshly isolated bone marrow cells were differentiated *in vitro* into macrophages as described earlier[25]. 2×10^4 cells were treated with trypsin, suspended in 2%

BMDM medium and seeded onto the upper chamber of an 8 μm polycarbonate filter (12-transwell format). Medium containing bevacizumab or deglycosylated bevacizumab (0.1 mg/ml), bevacizumab-Fab (0.067 mg/ml), bevacizumab-Fc (0.033 mg/ml), or human IgG1 (0.1 mg/ml) was placed in the lower chamber for 4 h prior to addition of mouse Vegfa (50 ng/ml; R&D Systems). After allowing cell migration for 12 h, cells were removed from the upper side of membranes, and nuclei of migratory cells on the lower side of the membrane were stained with Hoechst. The number of migratory cells was determined by fluorescence microscopy from the entire 32 mm diameter membrane (20× magnification of montage images) acquired using Cell Dimension Software. Cell numbers were acquired using ImageJ.

Cell stimulation and western blotting. Serum starved Py4 mouse blood endothelial cells were stimulated with 50 ng/ml of mouse Vegfa or human VEGFA for 10 min to assess Vegfr2 phosphorylation. 0.1 mg/ml bevacizumab (Genentech) or 0.75 μg/ml neutralizing anti-mouse Vegfa mAb (R&D Systems) were added to medium at the same time. Serum starved J774 mouse macrophages were stimulated with 0.1 mg/ml of bevacizumab or human IgG1 to assess c-Cbl phosphorylation. Serum starved RAW 264.7 mouse macrophages (ATCC, Manassus, VA) were stimulated with 0.1 mg/ml bevacizumab to assess Vegfr1 abundance. Bone marrow derived mouse macrophages (BMDMs) were prepared as previously described[26]. Bone marrow cells were cultured for 5 days in Iscove's modified Dulbecco's medium supplemented with 30% L929 cell supernatant containing sodium pyruvate, 10% heat-inactivated FBS (Life Technologies), non-essential Amino Acids (Sigma) and antibiotics. The differentiated BMDMs were serum starved and incubated with bevacizumab (0.1 mg/ml) for various time points. Human primary peripheral blood monocytes (Stemcell Technologies, Vancouver, Canada) and human monocytic

THP-1 cells (ATCC, authenticated by STR profiling) were serum starved in RPMI basal media for 4 h and then stimulated with 0.1 mg/ml of bevacizumab. Cells lysed in RIPA lysis buffer (Sigma-Aldrich) supplemented protease cocktail inhibitor were homogenized by sonication. Equal amounts of protein samples (20-40 μg) prepared in Laemmli buffer were resolved by SDS-PAGE on Novex® Tris-Glycine Gels (Life Technologies), and transferred onto Immun-Blot PVDF membranes (Bio-Rad Laboratories, Hercules, CA). The transferred membranes were blocked for 1 h at RT and incubated with antibodies against phospho-Vegfr2 (1:1000; Cell Signaling, Beverly, MA), Vegfr1 (1:1000; Abcam), phospho-c-Cbl (1:2000; Cell Signaling), phospho-c-CBL (1:2000; Abcam), or VEGFR1 (1:200, Santa Cruz Biotechnology, Dallas, TX) at 4 °C overnight. The immunoreactive bands were developed by enhanced chemiluminescence reaction. Protein loading was assessed by western blotting using antibodies against α-Tubulin (1:10000; Sigma-Aldrich), HSP70 (1:1000; Cell Signaling) or β–Actin (1:10,000; Sigma-Aldrich). Cultures routinely tested negative for mycoplasma.

Mouse IgG2c and human IgG1 ELISAs. Mouse blood was collected from C57BL/6J and *Rag2*$^{-/-}$ mouse jugular veins and serum was prepared. Corneas and retinae were harvested from C57BL/6J mice following perfusion with pre-warmed 1× PBS (40 mL) with heparin (20 U/mL) via the left ventricle to remove blood from the body using a 27-G needle. Corneas were chopped into fine pieces. Cornea and retina samples were homogenized by sonication. Samples were spun at 14,000×g for 20 min at 4 °C and the supernatant was transferred to a clean tube. Tissue supernatant was used to perform protein assays. Mouse IgG2c (Abcam) and human IgG1 (Abcam) ELISAs were performed according to the manufacturer's instructions. Measurements

were performed using Synergy 4 Microplate Reader and Gene5 software (BioTec, Suffolk, United Kingdom).

FcγRI interaction and phosphorylation *in vivo*. Bevacizumab and denosumab were biotinylated using the EZ-Link Sulfo-NHS-LC-Biotinylation Kit (Thermo Scientific) by following the manufacturer's instructions. After the reaction, by using the HABA biotin assay (Pierce Biotin Quantitation Kit; Thermo Scientific), it was found that each protein molecule had been modified by ~ 3 biotin molecules. Biotinylated bevacizumab or denosumab (25 mg/ml; 4 μl) was injected into wild-type mouse corneas immediately following suture injury. Two days later, corneas were excised and cell lysates were prepared by pooling 4 corneas in each group. Equal amounts of total corneal protein (400 ug) from each group were subjected to "pull-down" with Dynabeads® M-280 Streptavidin (Life Technologies) for 2 h at RT and eluted it by 1X protein sample buffer for western blotting. The blot was probed with anti-mouse Fcgr1 antibody (clone X54-5/7.1, BioLegend, San Diego, CA) overnight at 4 °C. The same blot was reprobed with HRP-conjugated Avidin (BioLegend). Bevacizumab (25 mg/ml; 4 μl) or PBS was injected into FcγR humanized mouse corneas immediately following suture injury. Two days later, corneas were excised and cell lysates were prepared by pooling 8 corneas in each group. Equal amounts of total corneal protein (300 μg) from each group were pre-cleared with Protein A/G Magnetic Beads (Pierce Classic Magnetic IP/Co-IP Kit, Thermo Scientific) for 1 h at RT to remove non-specific binding proteins. The pre-cleared samples were incubated with 10 μg of anti-human FcγRI antibody (clone 32.2, Acris Antibodies, San Diego, CA) overnight at 4 °C with gentle shaking. The immune complex was then precipitated with Protein A/G Magnetic Beads for 2 h at 4 °C, collected by magnets and washed three times with ice-cold IP Lysis/Wash

Buffer and once with purified water, and eluted by 1× protein sample buffer for western blotting. The blot was reprobed with anti-human FcγRI antibody to assess equal loading. The blot was probed with anti-phosphotyrosine antibody (Cell Signaling).

c-Cbl phosphorylation *in vivo*. Bevacizumab (25 mg/ml; 4 μl), bevacizumab-Fab (16 mg/ml; 4 μl), or bevacizumab-Fc (8 mg/ml; 4 μl) was injected into wild-type mouse corneas immediately following suture injury. Two days later, corneas were excised and cell lysates were prepared by pooling 4 corneas in each group. Equal amounts of protein samples (20–40 μg) prepared in Laemmli buffer were resolved by SDS-PAGE on Novex® Tris-Glycine Gels (Life Technologies), and transferred onto Immun-Blot PVDF membranes (Bio-Rad Laboratories). The transferred membranes were blocked with 3% BSA in TBS-T for 2 h at 4 °C and incubated with antibodies against phospho-c-Cbl (1:500; Cell Signaling) at 4 °C overnight. The immunoreactive bands were developed by enhanced chemiluminescence reaction. Protein loading was assessed by western blotting using antibodies against α-Tubulin (1:10000; Sigma-Aldrich).

Dynamic light scattering. Bevacizumab (25 mg/ml) in BBS buffer (10 mM Sodium Borate, pH 8.2, 150 mM NaCl) was treated at 63 °C for 1 h to induce aggregation. The size distributions of native and heat-treated bevacizumab were measured using Dynamic Light Scattering (Zetasizer Nano-ZS, Malvern Instruments, Malvern, United Kingdom).

Statistical analyses. Choroidal angiogenesis volumes per laser lesion were compared by hierarchical logistic regression using repeated measures analysis as previously described[11,12]. We used the Mann-Whitney *U* test with Bonferroni correction for statistical comparison of multiple

variables. Results are expressed as mean ± s.e.m. Type-I error not exceeding 0.05 was deemed significant.

Notes

1. Botto, M. *et al.* Homozygous C1q deficiency causes glomerulonephritis associated with multiple apoptotic bodies. *Nature genetics* **19**, 56-59 (1998).

2. O'Keefe, T. L., Williams, G. T., Davies, S. L. & Neuberger, M. S. Mice carrying a CD20 gene disruption. *Immunogenetics* **48**, 125-132 (1998).

3. Yamaguchi, R. *et al.* Cd52, known as a major maturation-associated sperm membrane antigen secreted from the epididymis, is not required for fertilization in the mouse. *Genes to cells : devoted to molecular & cellular mechanisms* **13**, 851-861 (2008).

4. Murphy, M. A. *et al.* Tissue hyperplasia and enhanced T-cell signalling via ZAP-70 in c-Cbl-deficient mice. *Molecular and cellular biology* **18**, 4872-4882 (1998).

5. Thien, C. B. *et al.* Loss of c-Cbl RING finger function results in high-intensity TCR signaling and thymic deletion. *EMBO J* **24**, 3807-3819 (2005).

6. Smith, P., DiLillo, D. J., Bournazos, S., Li, F. & Ravetch, J. V. Mouse model recapitulating human Fcgamma receptor structural and functional diversity. *Proc Natl Acad Sci U S A* **109**, 6181-6186 (2012).

7. Oettgen, H. C. *et al.* Active anaphylaxis in IgE-deficient mice. *Nature* **370**, 367-370 (1994).

8. Ioan-Facsinay, A. *et al.* FcgammaRI (CD64) contributes substantially to severity of arthritis, hypersensitivity responses, and protection from bacterial infection. *Immunity* **16**, 391-402 (2002).

9. Nimmerjahn, F. *et al.* FcgammaRIV deletion reveals its central role for IgG2a and IgG2b activity in vivo. *Proc Natl Acad Sci U S A* **107**, 19396-19401 (2010).

10. Mancardi, D. A. *et al.* FcgammaRIV is a mouse IgE receptor that resembles macrophage FcepsilonRI in humans and promotes IgE-induced lung inflammation. *The Journal of clinical investigation* **118**, 3738-3750 (2008).

11. de Haij, S. *et al.* In vivo cytotoxicity of type I CD20 antibodies critically depends on Fc receptor ITAM signaling. *Cancer Res* **70**, 3209-3217 (2010).

12. Gerber, H. P. *et al.* Mice expressing a humanized form of VEGF-A may provide insights into the safety and efficacy of anti-VEGF antibodies. *Proc Natl Acad Sci U S A* **104**, 3478-3483 (2007).

13. Kaneko, H. *et al.* DICER1 deficit induces Alu RNA toxicity in age-related macular degeneration. *Nature* **471**, 325-330 (2011).

14. Riechmann, L., Clark, M., Waldmann, H. & Winter, G. Reshaping human antibodies for therapy. *Nature* **332**, 323-327 (1988).

15. Armour, K. L., Clark, M. R., Hadley, A. G. & Williamson, L. M. Recombinant human IgG molecules lacking Fcgamma receptor I binding and monocyte triggering activities. *Eur J Immunol* **29**, 2613-2624 (1999).

16. Marino, M., Ruvo, M., De Falco, S. & Fassina, G. Prevention of systemic lupus erythematosus in MRL/lpr mice by administration of an immunoglobulin-binding peptide. *Nat Biotechnol* **18**, 735-739 (2000).

17. Albuquerque, R. J. *et al.* Alternatively spliced vascular endothelial growth factor receptor-2 is an essential endogenous inhibitor of lymphatic vessel growth. *Nat Med* **15**, 1023-1030 (2009).

18. Tarallo, V. *et al.* Inhibition of choroidal and corneal pathologic neovascularization by Plgf1-de gene transfer. *Invest Ophthalmol Vis Sci* **53**, 7989-7996 (2012).

19. Kleinman, M. E. *et al.* Sequence- and target-independent angiogenesis suppression by siRNA via TLR3. *Nature* **452**, 591-597 (2008).

20. Takeda, A. *et al.* CCR3 is a target for age-related macular degeneration diagnosis and therapy. *Nature* **460**, 225-230 (2009).

21. Cho, W. G. *et al.* Small interfering RNA-induced TLR3 activation inhibits blood and lymphatic vessel growth. *Proc Natl Acad Sci U S A* **106**, 7137-7142 (2009).

22. Lehr, H. A., Mankoff, D. A., Corwin, D., Santeusanio, G. & Gown, A. M. Application of photoshop-based image analysis to quantification of hormone receptor expression in breast cancer. *J Histochem Cytochem* **45**, 1559-1565 (1997).

23. Lehr, H. A., van der Loos, C. M., Teeling, P. & Gown, A. M. Complete chromogen separation and analysis in double immunohistochemical stains using Photoshop-based image analysis. *J Histochem Cytochem* **47**, 119-126 (1999).

24. Sakurai, E., Anand, A., Ambati, B. K., van Rooijen, N. & Ambati, J. Macrophage depletion inhibits experimental choroidal neovascularization. *Invest Ophthalmol Vis Sci* **44**, 3578-3585 (2003).

25. McDevitt, M. A. *et al.* A critical role for the host mediator macrophage migration inhibitory factor in the pathogenesis of malarial anemia. *J Exp Med* **203**, 1185-1196 (2006).

26. Franchi, L. *et al.* Cytosolic flagellin requires Ipaf for activation of caspase-1 and interleukin 1beta in salmonella-infected macrophages. *Nat Immunol* **7**, 576-582 (2006).
